# CRP Deficiency Rescues Periodontitis‐Induced Hippocampal Neurogenesis Impairment by Suppressing OPC‐Derived BMP4 Signaling in Rats

**DOI:** 10.1002/advs.202516199

**Published:** 2026-03-17

**Authors:** Lingjie Li, Ping Deng, Siyu Hou, Xianbo Xia, Wei Zhao, Xingyu Zhu, Yang Zhang, Chao Wang, Ling Xu, Jinlin Song

**Affiliations:** ^1^ College of Stomatology Chongqing Medical University Chongqing China; ^2^ Stomatological Hospital of Chongqing Medical University Chongqing China; ^3^ Chongqing Municipal Key Laboratory for Oral Biomedical Engineering of Higher Education Chongqing China; ^4^ College of Stomatology Xi'an Jiaotong University Xian China; ^5^ Key Laboratory of Biomechanics and Mechanobiology (Beihang University) Ministry of Education Key Laboratory of Innovation and Transformation of Advanced Medical Devices Ministry of Industry and Information Technology National Medical Innovation Platform for Industry‐Education Integration in Advanced Medical Devices (Interdiscipline of Medicine and Engineering) School of Biological Science and Medical Engineering School of Engineering Medicine Beihang University Beijing China

**Keywords:** bone morphogenetic protein 4, C‐reactive protein, hippocampus, oligodendrocyte progenitor cells, periodontal disease

## Abstract

Chronic periodontitis (PD) is an emerging risk factor for mental disorders, although the underlying mechanisms remain unclear. Here, we show that silk ligature‐induced PD in rats leads to anxiety/depression‐like behaviors and impaired hippocampal neurogenesis. These alterations were associated with elevated hippocampal levels of C‐reactive protein (CRP), derived predominantly from the periphery. Knockout of *Crp* attenuated these PD‐induced hippocampal impairments, largely through downregulation of bone morphogenetic protein 4 (*Bmp4*). Single‐nucleus RNA sequencing (snRNA‐seq) analysis revealed that hippocampal *Bmp4* expression is predominantly expressed by oligodendrocyte precursor cells (OPCs). Subsequent analyses associated CRP deficiency with enhanced functional resilience of OPCs. Critically, inhibition of the CRP‐BMP4 axis restored the neuronal differentiation suppressed by PD and alleviated the associated behavioral abnormalities. These findings elucidate a novel pathway linking periodontal disease to central nervous system disorders and suggest the CRP‐BMP4 axis as a potential therapeutic target for preventing PD‐related neurological complications.

## Graphical abstract

Chronic periodontitis elevates circulating CRP, which enters the hippocampus to upregulate BMP4 in oligodendrocyte precursor cells (OPCs), thereby impairing neurogenesis and inducing anxiety/depression‐like behaviors in rats. Counteracting this pathway, CRP deficiency helps confer functional resilience to OPCs. Importantly, by suppressing BMP4, CRP deficiency restores neuronal differentiation and alleviates periodontitis‐related behavioral abnormalities.

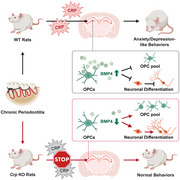

## 1 Introduction

Mental health disorders such as anxiety and depression represent significant causes of global disability [1, 2]. The current view frames anxiety and depression as being associated not only with social stress but also with inflammation and immune system activation. Inflammatory conditions, even those outside the central nervous system (CNS), can trigger or worsen mental disorder symptoms [3, 4]. In alignment with this evolving perspective, recent studies suggest that chronic periodontitis (PD), a condition characterized by its progressive and irreversible inflammatory nature, not only damages periodontal supporting structures and leads to tooth loss, but may also represent a potential risk factor for mental disorders [5]. The precise mechanisms underlying this association, however, require further investigation to fully elucidate the link between periodontal health and mental well‐being.

The progression of anxiety and depression involves a complex interplay among diverse cell types. Among these, neuronal and glial cells have emerged as a primary focus of investigation due to their profound involvement in these disorders [2, 6, 7]. Neuronal cells are the fundamental units of the nervous system. Recent studies have uncovered that the detrimental effects of PD on neurons primarily involve three aspects. On one side, PD‐related microbes and their products can either exacerbate or directly trigger the loss and degeneration of mature neurons [8, 9, 10, 11]. On the other side, bacteria such as *Porphyromonas gingivalis* (Pg) diminish the population of newly generated neurons [12]. Moreover, PD can also lead to synapse pruning and loss [12, 13].

Glial cells are crucial for supporting and maintaining neuronal function [7]. Accumulating evidence suggests that glial cells are susceptible to the impacts of PD and the pathogenic microbes that accompany it. Astrocyte numbers increase in the mouse hippocampus with ligature‐induced PD [14], as well as in mice treated with outer membrane vesicles or lipopolysaccharide (LPS) from Pg [15, 16]. Additionally, the regression of inflammation in periodontal tissues is accompanied by a decrease in the number of certain astrocyte subtypes in the hippocampus [14]. Similar studies also show pathological changes in microglia, including activated microglia and increased inflammation‐related factors, such as tumor necrosis factor‐α (TNF‐α) and interleukin‐1β (IL‐1β) [17, 18, 19]. However, despite the important role that oligodendrocyte precursor cells (OPCs) and oligodendrocytes play in anxiety and depression, which includes their involvement in myelination, neurotransmitter transmission, and neuroimmune regulation [19, 20], their potential connection to PD in the context of these disorders remains largely unexplored. As of the completion of this study, there is a notable absence of reports addressing this interesting intersection.

As research deepens, there is a growing recognition that the complex communication between glial cells and neuronal cells is a fundamental aspect in understanding mental disorders [7]. In addition to causing direct neuronal damage via glial‐derived inflammatory factors, Pg can also trigger synapse loss by activating the phagocytic function of microglia [12, 13]. Furthermore, Pg‐activated astrocytes can inhibit the maturation of BDNF, a key regulator of neuronal regeneration and synaptic plasticity [16]. However, research on PD‐influenced cellular interactions remains limited.

This study aimed to investigate the potential pathways through which PD contributes to the anxiety/depression‐like changes in the hippocampus. We established the Stress model using chronic restraint stress and the PD model using the silk ligature method. Behavioral tests indicated that PD induced anxiety/depression‐like behavior in rats. Immunohistochemical (IHC) staining revealed that PD reduced the immature neuron numbers in the hippocampus. Based on antibody arrays and single‐nucleus RNA sequencing (snRNA‐seq) results, we identified that the peripheral C‐reactive protein (CRP) is involved in PD‐induced behavioral changes. Using *Crp* knockout (KO) rats, we demonstrated that CRP deficiency effectively prevented these PD‐induced changes. Subsequent mRNA sequencing (mRNA‐seq) analysis revealed that CRP acts as an upstream regulator of bone morphogenetic protein 4 (*Bmp4*), with its hippocampal expression significantly decreased following *Crp* KO. Adeno‐associated virus (AAV)‐mediated overexpression of *Bmp4* reversed the protective effects of *Crp* KO in PD rats. Most critically, we found that OPCs are the primary sources of BMP4, whose upregulation led to a reduction in OPC numbers. Although CRP deficiency did not reverse this quantitative loss, it likely preserved myelination by enhancing the functional adaptability of the remaining OPC pool. Furthermore, we preliminarily investigated the role of hippocampal OPCs in neurogenesis in PD rats. The results demonstrated that PD impaired neuronal differentiation, as evidenced by reduced NeuroD1 expression, whereas *Crp* KO rescued this impairment by downregulating *Bmp4* in OPCs. In conclusion, this study identifies the CRP‐BMP4 axis as an important modulator of PD‐induced anxiety/depression‐like changes through its regulation of OPC‐derived BMP4 signaling and neuronal differentiation.

## 2 Results

### 2.1 PD Induces Anxiety/Depression‐Like Behaviors, Hippocampal Neurogenesis Reduction, and CRP Upregulation in Rats

**FIGURE 1:**
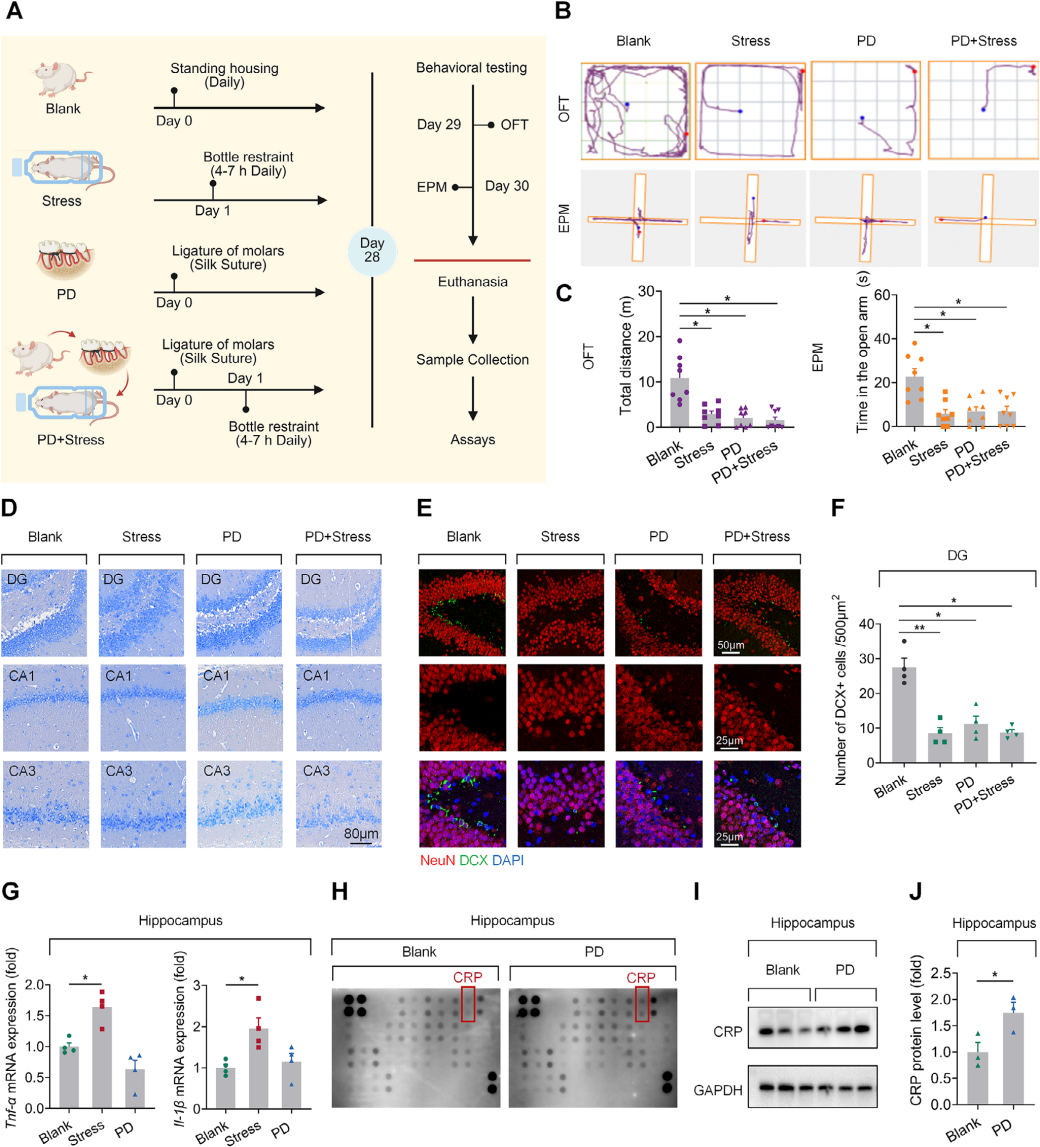
Silk ligature‐induced PD in rats shows behavioral and hippocampal changes. (A) Experimental timeline for disease modeling and behavioral testing: Chronic periodontitis (PD) was induced using silk ligatures around molars. Chronic stress (Stress) was modeled through restricted movement using water bottles, while the PD+Stress group received both interventions. Behavioral tests, including open field (OFT) and elevated plus maze (EPM), were performed after 4 weeks of modeling, followed by tissue collection. (B) Representative images of rat movement traces in the OFT and EPM. (C) Statistical analysis of OFT total distance and EPM open‐arm time. (D) Representative Nissl staining images of the DG, CA1, and CA3 areas. The Nissl bodies of normal neurons appear as dark blue granules. (E) Representative IHC staining images of mature neurons (NeuN^+^, red) and immature neurons (DCX^+^, green) in the DG area. DAPI (blue) marks the nuclei. (F) Statistical analysis of DCX^+^ immature neuron numbers in the DG area. (G) Statistical analysis of hippocampal *Tnf‐α* and *Il‐1β* mRNA expression by real‐time qPCR. (H) Inflammation antibody arrays of hippocampal lysates (red box highlights CRP). (I) Representative Western blot images of CRP in the hippocampus. (J) Statistical analysis of hippocampal CRP levels. Results are presented as mean ± SEM (*n* = 8 rats per group for C; *n* = 4 rats per group for F, G, and J). Statistical significance (^*^ *p* < 0.05, ^**^ *p* < 0.01) was assessed with one‐way ANOVA for C, F, and G and with an unpaired *t*‐test for J.

To investigate the link between PD and mental health disorders in rats, we induced PD (PD group) by placing silk ligatures around molars. Meanwhile, we constructed chronic mild stress models (Stress group) by restraining rats, using them as a comparator model for assessing anxiety/depressive symptoms. Furthermore, to determine whether PD exacerbates the behavioral and neural changes associated with chronic stress, we developed a combined model (PD+Stress group) with silk ligature placement and restraint (Figure 1A). After 28 days of modeling, we conducted behavioral tests including the Open Field Test (OFT) and the Elevated Plus Maze (EPM). Results showed that rats with stress or PD walked shorter distances in the OFT and spent less time in the open arms of the EPM compared to healthy rats (Blank group), indicating reduced exploration of unknown environments and increased avoidance of exposed spaces. Therefore, rats with PD exhibited behaviors similar to stress‐mediated anxiety/depression‐like behaviors. However, PD did not appear to further worsen the anxiety/depression‐like behavior in stressed rats (Figure 1B,C).

Upon observing the behavioral changes, we further focused on the hippocampus, an area closely linked to mental disorders like anxiety and depression. Nissl and IHC staining revealed that both PD and stress disturbed the orderly arrangement of neurons in the DG, CA1, and CA3 areas (Figure 1D,E). Additionally, the number of doublecortin (DCX)^+^ immature neurons decreased by approximately half (Figure 1E,F). These data suggest that PD may affect neurogenesis. In line with the behavioral test outcomes, the combined effects of PD and stress did not appear to exacerbate the neuronal impairment further (Figure 1D–F).

The prevailing view suggests that anxiety/depression‐like changes in the brain often accompany inflammation. Consequently, we tested the mRNA expression of proinflammatory factors within the hippocampus. Real‐time quantitative PCR (real‐time qPCR) analysis revealed that stress upregulated the expression of *Tnf‐α* and *Il‐1β* in the hippocampus compared to healthy control rats. However, this up‐regulation was not evident in PD rats (Figure 1G). This suggests that the hippocampal pathology induced by PD (via silk ligature placement) differs partially from that caused by chronic stress. To find the potential mechanisms by which PD affects hippocampal pathology, we employed inflammation antibody arrays to preliminarily screen for changes in inflammation‐related proteins caused by PD. CRP was identified as one of the proteins upregulated in the PD group, as compared to the healthy control group (Figure 1H). This finding was further validated through Western blot analysis (Figure 1I,J).

### 2.2 *Crp* KO Alleviates PD‐Induced Hippocampal Neurogenesis Reduction and Behavioral Changes

**FIGURE 2:**
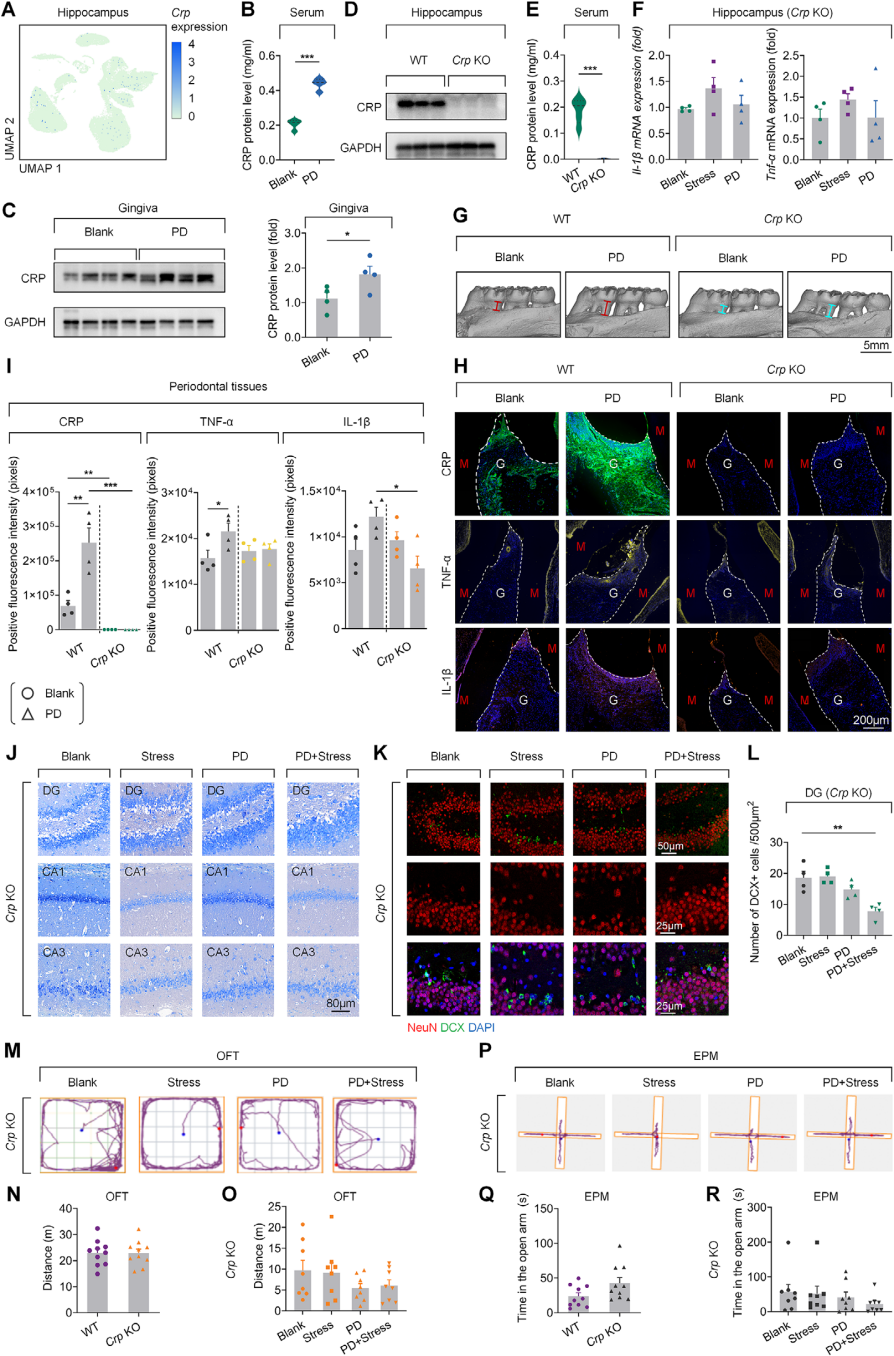
*Crp* KO attenuates PD‐induced hippocampal neurogenesis impairment and behavioral alterations. (A) snRNA‐seq analysis of *Crp* expression across hippocampal cell subpopulations. The sequencing library was constructed from a pool of hippocampal tissues from 12 rats. All downstream analyses and visualizations are presented at the single‐nucleus level. Color gradient (blue: high; green: low) represents relative *Crp* expression levels. (B) Peripheral blood CRP levels in WT rats with/without PD by ELISA. (C) Representative Western blot images and statistical analysis of gingival CRP in WT rats with or without PD. (D) Representative Western blot images of gingival CRP in unstimulated WT and *Crp* KO rats. (E) Peripheral blood CRP levels in unstimulated WT and *Crp* KO rats. (F) Statistical analysis of hippocampal *Tnf‐α* and *Il‐1β* mRNA expression by real‐time PCR in *Crp* KO rats with/without PD and stress. (G) Representative three‐dimensional micro‐CT reconstruction images of alveolar bone. Red and blue lines demarcate alveolar bone height from cementoenamel junction to crest in WT and *Crp* KO rats respectively (PD groups shown as dashed lines). (H) Representative IHC staining images of periodontal CRP (green), TNF‐α (yellow), and IL‐1β (red) in WT and *Crp* KO rats with or without PD. DAPI (blue) marks the nuclei. M, molar; G, gingiva. (I) Statistical analysis of CRP, TNF‐α, and IL‐1β fluorescence intensity, respectively. (J) Representative Nissl staining images of the DG, CA1, and CA3 areas in *Crp* KO rats under Blank, Stress, PD, and PD+Stress. (K) Representative IHC staining images of mature neurons (NeuN^+^, red) and immature neurons (DCX^+^, green) of the DG area. DAPI (blue) marks the nuclei. (L) Statistical analysis of DCX^+^ immature neuron numbers in the DG area. (M–R) Behavioral analysis. Representative images of movement traces of *Crp* KO rats under Blank, Stress, PD, and PD+Stress in OFT (M) and EPM (P). Statistical analysis of OFT total distance (N) and EPM open‐arm time (Q) of unstimulated WT or *Crp* KO rats. Statistical analysis of OFT total distance (O) and EPM open‐arm time (R) of *Crp* KO rats under Blank, Stress, PD, and PD+Stress. Results are presented as mean ± SEM (*n* = 8 rats per group for B, O, and R; *n* = 4 rats per group for C, E, F, I, and L; *n* = 10 rats per group for N and Q). Statistical significance (^*^ *p* < 0.05, ^**^ *p* < 0.01, ^***^ *p* < 0.001) was assessed with one‐way ANOVA for F, I, L, O, and R and with an unpaired *t*‐test for B, C, E, N, and Q.

To explore whether and how CRP participates in PD‐related anxiety/depression‐like changes, we first utilized snRNA‐seq to identify *Crp*‐expressing cells in the hippocampus. The data revealed, however, that hippocampal cell populations do not appear to be the primary producers of CRP (Figure 2A). Given that recent studies have indicated that circulating CRP is associated with the risk of mental disorders [21, 22], we hypothesized that the increased hippocampal CRP levels in PD rats might originate from extracranial sources. To test this hypothesis, we measured peripheral CRP levels by ELISA, which showed significant elevation in PD rats (Figure 2B). Notably, this systemic increase coexisted with upregulated CRP in the gingiva (Figure 2C). Since impaired blood‐brain barrier (BBB) integrity represents a potential route for circulating proteins to enter the brain, we further assessed the expression of BBB integrity markers: zonula occludens‐1 (ZO‐1), a critical tight junction component regulating paracellular permeability, and caveolin‐1 (CAV‐1), a principal mediator of transcytosis. IHC staining revealed no significant differences in the fluorescence intensity of ZO‐1 or CAV‐1 in the hippocampus between the PD and Blank groups (Figure S1). These results suggest that the PD‐induced elevation of hippocampal CRP does not rely on changes in the expression of ZO‐1 or CAV‐1, and the underlying mechanism requires further investigation.

Then we utilized TALEN‐generated *Crp* KO rats to investigate the role of CRP in PD‐induced anxiety/depression‐like changes. Knockout efficiency was confirmed by measuring CRP protein levels in both hippocampus and peripheral blood of wild‐type SD (WT) and *Crp* KO rats (Figure 2D,E). We then established PD and Stress models using KO rats. Real‐time qPCR analysis revealed that PD did not alter hippocampal *Tnf‐α* and *Il‐1β* mRNA expression in the *Crp* KO rats (Figure 2F). Notably, CRP deficiency inhibited the stress‐induced upregulation of these proinflammatory cytokines in WT rats (Figure 1G,F). Although not the primary focus of this study, this phenomenon provides evidence for the involvement of CRP in stress‐related neuroinflammation. Furthermore, the IHC staining results showed that PD upregulated the protein levels of periodontal CRP and TNF‐α. Although not statistically significant, IL‐1β levels also exhibited an upward trend. Additionally, *Crp* KO reduced periodontal inflammation. (Figure 2G–I).

Next, we explored whether CRP deficiency could alleviate the anxiety/depression‐like changes. Compared to unstimulated *Crp* KO rats, those exposed to PD or stress showed no significant difference in the arrangement of mature neurons within the DG, CA1, and CA3 areas (Figure 2J,K). Likewise, no significant difference was observed in the number of DCX^+^ immature neurons (Figure 2K,L). However, under PD+Stress conditions, the protective effect of *Crp* KO on neurogenesis was attenuated, as it failed to prevent the reduction of immature neurons (Figure 2K,L). Subsequently, we conducted behavioral tests. Results revealed that *Crp* KO did not significantly affect the total walking distance of rats in EPM (Figure 2M,N) or the time spent in the open arms of OFT (Figure 2P,Q). Importantly, PD induction—with or without concurrent stress – failed to produce significant behavioral changes in *Crp* KO rats during both OFT (Figure 2M,O) and EPM tests (Figure 2P,R). These findings imply that CRP deficiency may protect against PD‐related anxiety/depression‐like changes.

### 2.3 *Crp* KO Alleviates PD‐Induced Hippocampal Impairments via BMP4 Signaling

**FIGURE 3:**
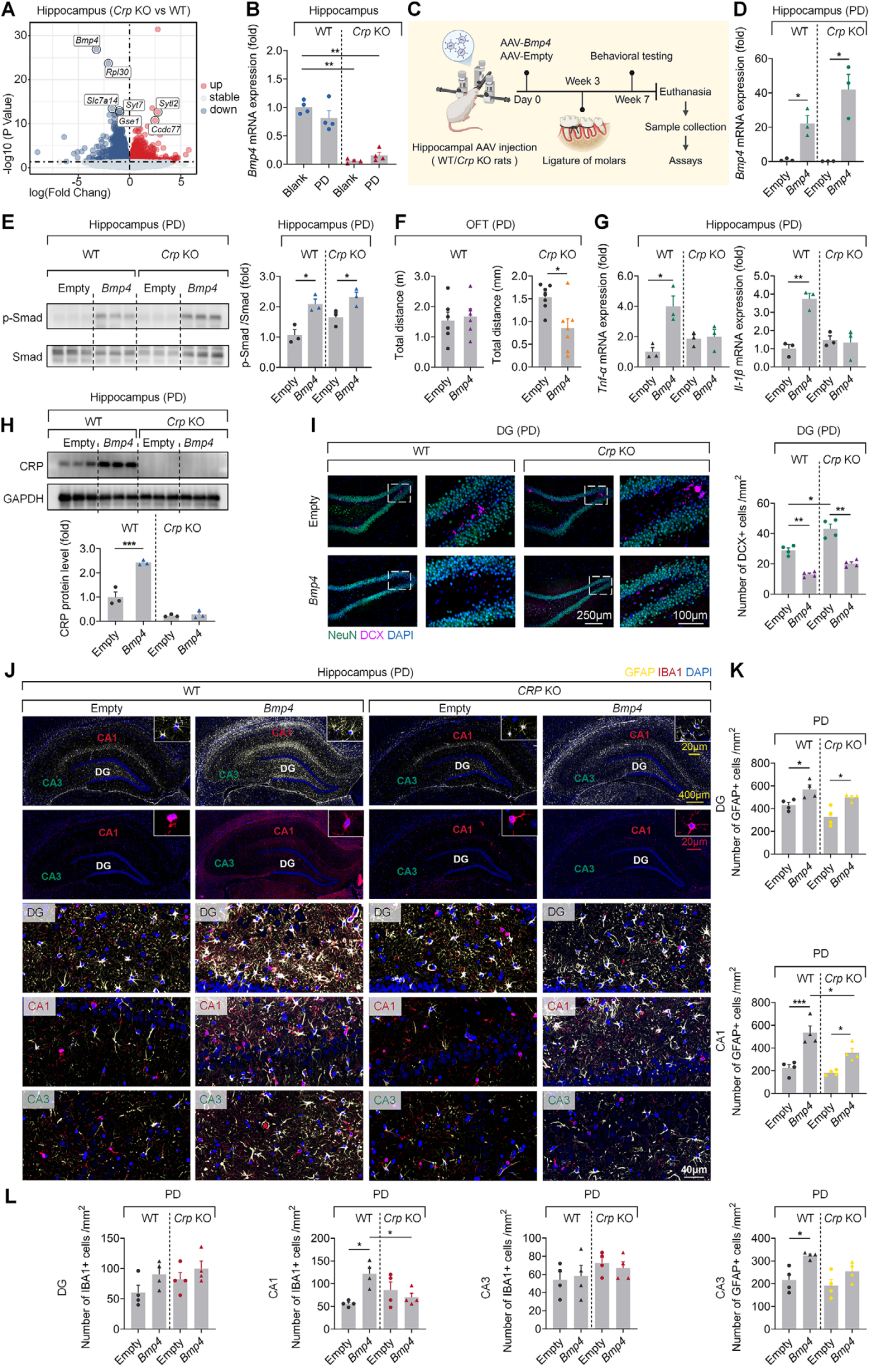
*Bmp4* overexpression counteracts the protective effects of *Crp* KO in PD rats. Collecting rat hippocampal tissues for mRNA‐seq. (A) Differential gene expression (DEGs) analysis of hippocampal tissues from unstimulated WT and *Crp* KO rats. 3–4 hippocampal tissue samples per group. (B) Statistical analysis of hippocampal *Bmp4* expression by real‐time qPCR in WT and *Crp* KO rats with or without PD. (C) Experimental timeline for AAV (Empty or *Bmp4*) hippocampal injection and PD modeling (WT and *Crp* KO rats): Hippocampal injections were performed at baseline, followed by PD induction via silk ligature at 3 weeks post‐injection. Behavioral test (OFT) was conducted at 4 weeks post‐ligature, with subsequent tissue collection. (D) Statistical analysis of hippocampal *Bmp4* expression by real‐time qPCR in AAV‐injected rats. (E) Representative Western blot images and statistical analysis of hippocampal p‐SMAD/SMAD in AAV‐injected rats. (F) Statistical analysis of OFT total distance of AAV‐injected WT (left) and *Crp* KO (right) rats. (G) Statistical analysis of hippocampal *Tnf‐α* and *Il‐1β* expressions by real‐time qPCR in AAV‐injected rats. (H) Representative Western blot images and statistical analysis of hippocampal CRP in AAV‐injected rats. (I) Representative IHC staining images of mature neurons (NeuN^+^, green) and immature neurons (DCX^+^, plum) in the hippocampal DG area of AAV‐injected rats. DAPI (blue) marks the nuclei; statistical analysis of immature neurons. (J) Representative IHC staining images of astrocytes (GFAP^+^, yellow) and microglia (IBA1^+^, red) in DG, CA1 and CA3 areas of AAV‐injected rats. Inserts in the top right corners of the first‐ and second‐row images show enlarged views of representative astrocytes and microglia, respectively. DAPI (blue) marks the nuclei. (K, L) Statistical analysis of astrocytes and microglia numbers. Results are presented as mean ± SEM (*n* = 4 rats per group for B, G, I, K, and L; *n* = 3 rats per group for D, E, and H; *n* = 7 rats per group for F). Statistical significance (^*^ *p* < 0.05, ^**^ *p* < 0.01, ^***^ *p* < 0.001) was assessed with one‐way ANOVA for B, G, I, K, and L and with an unpaired *t*‐test for F.

After observing that CRP deficiency alleviates anxiety/depression‐like behaviors and neurogenesis impairment in PD rats, we further investigated its potential mechanism. We began by performing mRNA‐seq of hippocampal samples from WT and *Crp* KO rats. Principal component analysis (PCA) confirmed sample repeatability (Figure S2). Differential gene expression (DEGs) analysis revealed 1622 genes upregulated and 2013 downregulated genes in the hippocampus of the *Crp* KO rats. Notably, *Bmp4* showed the most significant downregulation of the *Crp* KO rats (Figure 3A), a finding further validated by real‐time qPCR (Figure 3B).

Previous studies have suggested that BMP signaling may serve as a potential therapeutic for mental disorders like depression [23]. Therefore, while the real‐time qPCR results did not detect significant PD‐induced *Bmp4* upregulation in whole hippocampal tissue (Figure 3B), we hypothesized that the CRP deficiency might exert its hippocampal protective effects through *Bmp4* downregulation in the hippocampus. To investigate this, we constructed an AAV to overexpress *Bmp4* (AAV‐*Bmp4*) and injected it into the hippocampus of both WT and *Crp* KO rats. After 3 weeks, we induced the PD model (Figure 3C). 4 weeks later, analyses of the hippocampus using real‐time qPCR and Western blot revealed that AAV‐*Bmp4*, compared to empty vector controls (AAV‐Empty), successfully elevated *Bmp4* mRNA levels (Figure 3D) and enhanced phosphorylation of its downstream effector SMAD (Figure 3E). Notably, *Crp* KO failed to inhibit this AAV‐mediated BMP4 upregulation.

To determine whether increased BMP4 could counteract the protective effects of CRP deficiency in PD rats, we detected hippocampal inflammatory responses, behavior changes, and neurogenesis following AAV injection. In WT rats with PD, *Bmp4* overexpression did not further alter the total walk distance in the OFT (Figure 3F), but markedly elevated hippocampal *Il‐1β* and *Tnf‐α* mRNA levels (Figure 3G). Unexpectedly, it also increased CRP protein expression in WT rats with PD (Figure 3H), suggesting BMP4 may function as a pro‐inflammatory mediator that potentially regulates CRP bidirectionally. While *Crp* KO prevented the BMP4‐induced upregulation of pro‐inflammatory factors (Figure 3G), the KO rats exhibited reduced total walk distance in the OFT (Figure 3F). We next observed the influence of AAV‐*Bmp4* on neurogenesis. IHC images revealed that there were more immature neurons in the *Crp* KO rats with PD compared to the WT rats with the same condition. Notably, *Bmp4* overexpression significantly reduced immature neuron numbers in both WT and *Crp* KO rats (Figure 3I). These findings demonstrate that while BMP4 signaling abolishes the protective effects of CRP deficiency against both behavioral abnormalities and neurogenesis impairment in PD rats, CRP deficiency effectively attenuates BMP4‐induced neuroinflammation.

In addition to changes in neurogenesis, we examined the effects of BMP4 and CRP on astrocytes and microglia. IHC analysis revealed no significant differences in the number or morphology of astrocytes between WT and *Crp* KO rats, regardless of the presence of PD existed or not (Figure S3A,B,D). Similarly, microglial numbers and morphology were comparable across all groups under baseline conditions (Figure S3A,C,D). However, in WT rats with PD, *Bmp4* overexpression increased the number and morphological complexity of astrocytes (Figure 3J,K; Figure S4A) and selectively elevated microglial numbers in the CA1 area (Figure 3L). Although *Crp* KO did not completely block these responses, it attenuated the overexpress *Bmp4*‐induced increase in astrocyte numbers in the CA1 and CA3 areas, suppressed the associated astrocytic morphological changes, and prevented the rise in microglial numbers in CA1. Thus, while *Bmp4* overexpression altered astrocytes and microglia, the PD‐induced upregulation of the CRP‐BMP4 axis alone did not lead to significant changes in these cells within the duration of our model.

### 2.4 *Crp* KO Prevents PD‐Induced *Bmp4* Upregulation in Hippocampal OPCs

**FIGURE 4:**
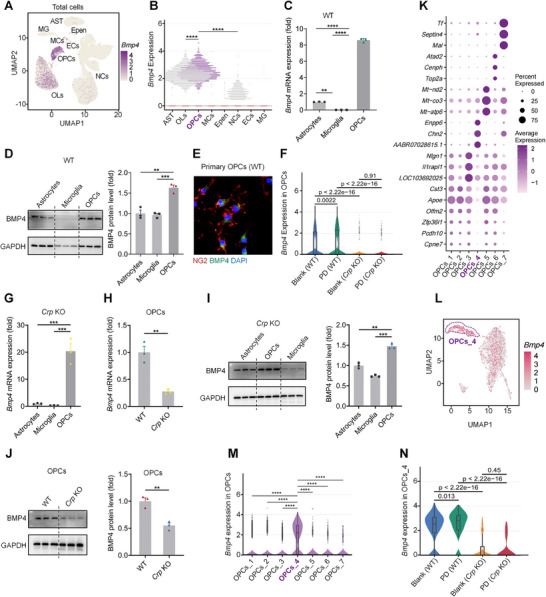
PD increases Bmp4 expression in hippocampal OPCs. (A, B, F, and K–N) snRNA‐seq analysis of hippocampal tissues from WT and Crp KO rats with or without PD. Libraries were constructed from pooled tissues (12 rats total; F and N used three rats per group). Data are visualized at the single‐nucleus level. Statistical significance (^***^ *p*<0.0001) was determined by the Wilcoxon rank‐sum test on aggregated single‐nucleus expression data. (C, D, and G–J) Analysis of primary astrocytes, microglia, and OPCs isolated from neonatal rat hippocampal tissues. Three independent biological replicates, each pooled from 4‐5 rats, were analyzed per group. Statistical significance was assessed by one‐way ANOVA (C, D, G, I; ^**^ *p*<0.01, ^***^ *p*<0.001) or unpaired t‐test (H, J). (A) UMAP visualization of hippocampal cell clusters. Identified cell populations included astrocytes (AST), oligodendrocytes (OLs), oligodendrocyte progenitor cells (OPCs), mural cells (MCs), ependymal cells (Epen), neuronal cells (NCs), endothelial cells (ECs), and microglia (MG). Color gradient (Purple: high; Sand: low) indicates *Bmp4* expression levels. (B) Comparative *Bmp4* expression across hippocampal cell types, derived from snRNA‐seq data. (C) Statistical analysis of *Bmp4* expression by real‐time qPCR in primary astrocytes, microglia, and OPCs from WT rats. (D) Representative Western blot images and statistical analysis of BMP4 in primary astrocytes, microglia, and OPCs from WT rats. (E) Representative immunofluorescence staining images of BMP4 (green) in primary OPCs (NG2^+^, red) from WT rats. DAPI (blue) marks the nuclei. (F) *Bmp4* expression in OPCs from WT and *Crp* KO rats with or without PD, derived from snRNA‐seq data. Significant adjusted *p* values are shown. (G) Statistical analysis of *Bmp4* expression by real‐time qPCR in primary astrocytes, microglia, and OPCs from *Crp* KO rats. (H) Statistical analysis of *Bmp4* expression by real‐time qPCR in primary OPCs from WT and *Crp* KO rats. (I) Representative Western blot images and statistical analysis of BMP4 in primary astrocytes, microglia, and OPCs from *Crp* KO rats. (J) Representative Western blot images and statistical analysis of BMP4 in primary OPCs from WT and *Crp* KO rats. (K) OPC subclustering analysis revealed 7 distinct populations (OPC_1‐7). The dashed purple circle highlights the *Bmp4*‐high subpopulation. Color gradient (pink: high; gray: low) represents relative *Bmp4* expression levels. (L) Dot plot displaying marker gene expression patterns in each OPC subgroup. Dot size represents the percentage of cells expressing the gene, derived from snRNA‐seq data. Dot color indicates the average expression level in expressing cells (purple: high). (M) *Bmp4* expression across OPC subtypes, derived from snRNA‐seq data. (N) *Bmp4* expression in OPCs_4 across WT and *Crp* KO rats with or without PD, derived from snRNA‐seq data. Significant adjusted *p* values are shown.

Our preliminary findings indicated that *Crp* KO‐mediated hippocampal protection in PD rats involved *Bmp4* downregulation. To elucidate how and where BMP4 worked in this process, we analyzed rat hippocampal snRNA‐seq data and identified major cell populations including astrocytes (AST), oligodendrocytes (OLs), oligodendrocyte progenitor cells (OPCs), mural cells (MCs), ependymal cells (Epen), neuronal cells (NCs), endothelial cells (ECs), and microglia (MG) (Figure 4A; Figure S5A). OPCs emerged as the primary *Bmp4*‐expressing population, followed by oligodendrocytes (Figure 4A,B). Hippocampal *Bmp4* ^+^ cell numbers were significantly reduced in *Crp* KO rats compared to WT rats, whether rats had PD or not (Figure S5B). We then isolated primary OPCs, astrocytes, and microglia (Figure S5C) from neonatal WT rat brains and confirmed high *Bmp4* expression in OPCs using real‐time qPCR (Figure 4C). Western blot and immunofluorescence analyses also detected BMP4 protein in OPCs (Figure 4D,E). We also examined *Bmp4* expression in neonatal *Crp* KO rat brains. Consistent with the snRNA‐seq data (Figure 4A,B,F), OPCs from *Crp* KO rats also expressed *Bmp4* (Figure 4G), though at lower levels than those in WT rats (Figure 4H). Similarly, Western blot detected BMP4 protein in OPCs from *Crp* KO rats, but at a reduced level compared to WT controls (Figure 4I,J). These results indicate that BMP4 is produced in OPCs and that CRP functions as an upstream regulator. Interestingly, while astrocytes showed low but detectable *Bmp4* expression in neonatal brains (Figure 4C,G), it was nearly undetectable in the adult hippocampus (Figure 4A,B), suggesting that the expression pattern of *Bmp4* may be age‐dependent.

Since OPCs showed the strongest *Bmp4* expression, we focused on analyzing their response to PD. While real‐time qPCR analysis of whole hippocampal tissue did not detect significant PD‐induced *Bmp4* upregulation (Figure 3B), analysis at single‐nucleus resolution revealed an increase in *Bmp4* expression within the OPC lineage in PD rats, an effect blocked by *Crp* KO (Figure 4F). Further unsupervised clustering identified seven transcriptionally distinct OPC subpopulations, designated OPCs_1 through OPCs_7 (Figure 4K,L; Figure S5D). OPCs_4 was identified as a subpopulation marked by high expression of intermediate oligodendrocyte differentiation markers (*Enpp6* and *Chn2*) (Figure 4K), and it showed the highest basal *Bmp4* expression among all subpopulations (Figure 4M). In OPCs_4, PD induced a further significant increase in *Bmp4* (Figure 4N), which was prevented by *Crp* KO. Collectively, these data demonstrate a CRP‐dependent upregulation of *Bmp4* in hippocampal OPCs following PD, notably within defined differentiation‐primed subpopulations such as OPCs_4.

### 2.5 The CRP‐BMP4 Axis Modulates Hippocampal OPC Homeostasis in PD Rats

**FIGURE 5:**
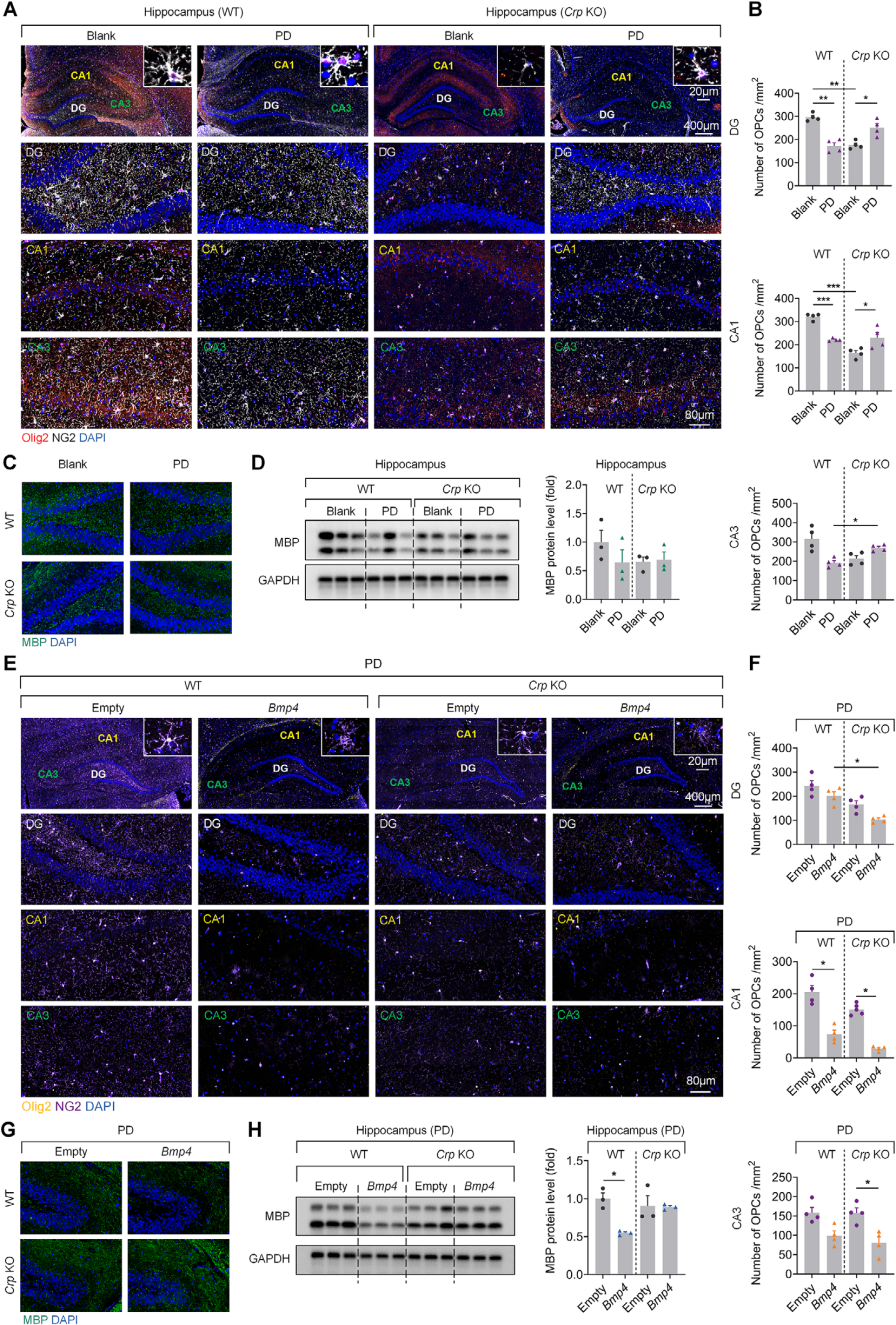
*Crp KO* preserves hippocampal OPC pool in PD rats, an effect counteracted by *Bmp4* overexpression. WT and *Crp* KO rats were subjected to PD induction or maintained as untreated controls (Blank group): (A) Representative IHC staining images of OLIG2^+^ (red) NG2^+^ (White) OPCs in hippocampal DG, CA1, and CA3 areas. Inserts in the top right corners of the first‐row images show enlarged views of representative OPCs. DAPI (blue) marks the nuclei. (B) Statistical analysis of OPC numbers across groups. (C) Representative IHC staining images of MBP (green) in the hippocampal DG area. DAPI (blue) marks the nuclei. (D) Representative Western blot images and statistical analysis of hippocampal MBP levels. WT and *Crp* KO rats injected with AAV‐Empty or AAV‐*Bmp4* were subjected to PD induction: (E) Representative IHC staining images of OLIG2^+^ (yellow) NG2^+^ (purple) OPCs in hippocampal DG, CA1 and CA3 areas. Inserts in the top right corners of the first‐row images show enlarged views of representative OPCs. DAPI (blue) marks the nuclei. (F) Statistical analysis of OPC numbers. (G) Representative IHC staining images of MBP (green) in the hippocampal DG area. DAPI (blue) marks the nuclei. (H) Representative Western blot images and statistical analysis of hippocampal MBP levels. Results are presented as mean ± SEM (*n* = 4 rats per group for B and F; *n* = 3 rats per group for D and E). Statistical significance (^*^ *p* < 0.05, ^**^ *p* < 0.01, ^***^ *p* < 0.001) was assessed with one‐way ANOVA.

We next examined the effects of PD and CRP on OPCs. Co‐immunostaining for neural/glial antigen 2 (NG2) and oligodendrocyte transcription factor 2 (OLIG2) revealed that PD reduced NG2^+^OLIG2^+^ OPC numbers in the hippocampus of WT rats (Figure 5A,B). Interestingly, while *Crp* KO rats had fewer OPCs than WT rats at baseline, PD increased OPC numbers in the DG and CA1 regions of *Crp* KO rats. Morphologically, most OPCs in CRP‐deficient hippocampus exhibited fewer branches (Figure 5A; Figure S6A), a phenotype consistent with a less differentiated state. However, neither IHC nor Western blot analysis detected significant differences in myelin basic protein (MBP), a mature oligodendrocyte marker, between WT and *Crp* KO rats, with or without PD (Figure 5C,D). These results suggest that although PD reduces OPC numbers in WT rats, this reduction does not substantially affect myelination. Furthermore, the OPC pool in *Crp* KO rats, while smaller and less differentiated, remains functionally competent to maintain myelin homeostasis even under PD conditions.

Subsequently, to investigate the role of the CRP‐BMP4 axis in hippocampal OPC homeostasis, we performed AAV‐mediated *Bmp4* overexpression and assessed its effect on OPC numbers and myelination. *Bmp4* overexpression significantly reduced OPC numbers in both WT and *Crp* KO rats under PD conditions (Figure 5E,F). Moreover, *Bmp4* overexpression induced potentially pathological alterations in OPC morphology, characterized by complex and disorganized protrusions in some OPCs of both WT and *Crp* KO rats (Figure 5E; Figure S6B). Importantly, IHC and Western blot analysis revealed that while *Bmp4* overexpression decreased MBP levels in WT rats, *Crp* KO rats maintained normal MBP levels despite *Bmp4* overexpression (Figure 5G,H). These results demonstrate that *Bmp4* overexpression exacerbates OPC loss and disrupts myelination in WT rats. In *Crp* KO rats, although *Bmp4* overexpression suppressed the PD‐induced increase in OPC numbers, it did not compromise myelination. This indicates that the protective effect of CRP deficiency on myelination can be maintained even when OPC numbers are low, as seen under *Bmp4* overexpression, and may involve mechanisms beyond the regulation of BMP4.

### 2.6 The CRP‐BMP4 Axis Regulates Hippocampal Neuronal Differentiation in PD Rats

**FIGURE 6:**
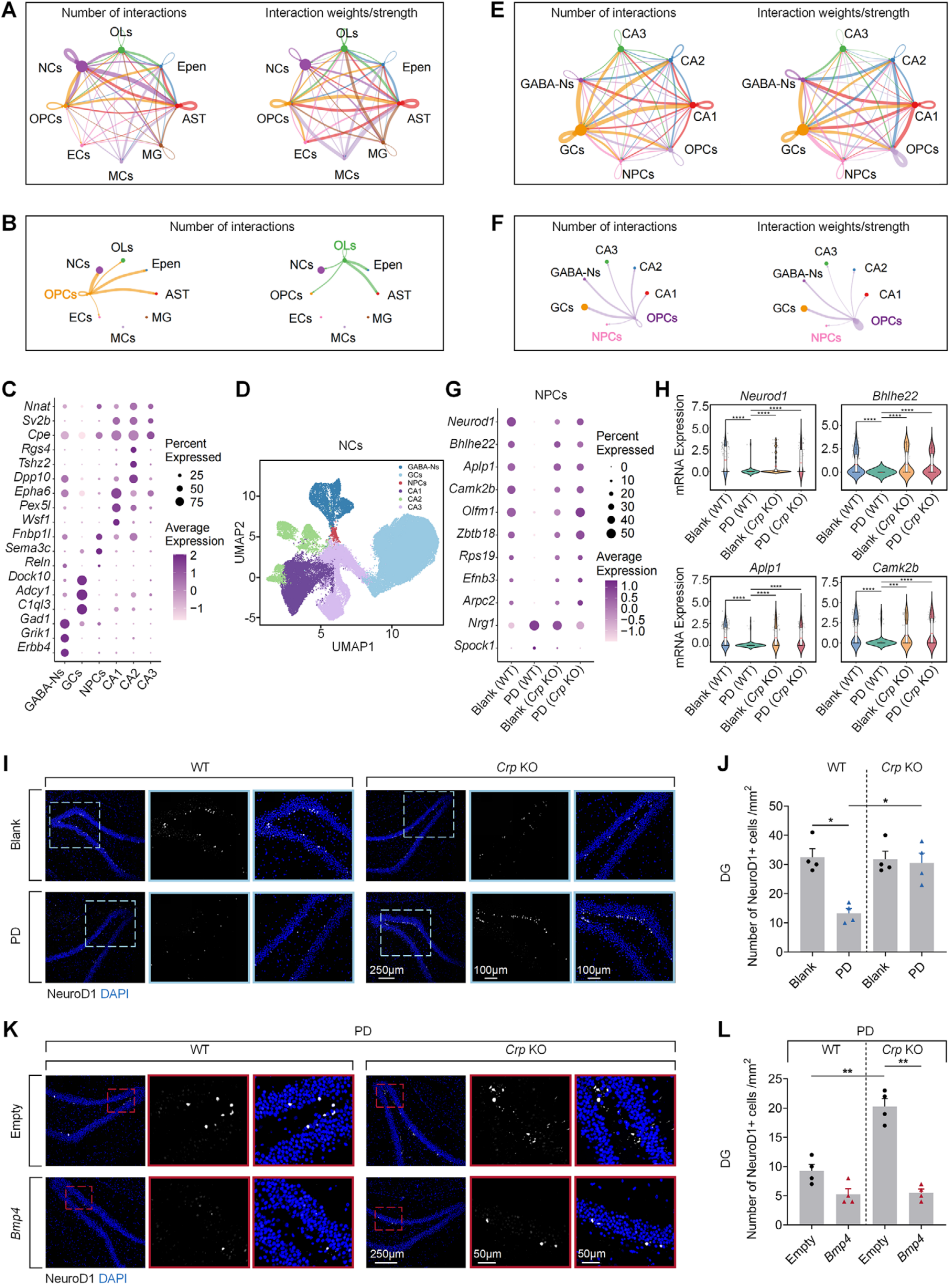
*Crp* KO rescues PD‐impaired NPC differentiation, an effect reversed by BMP4 overexpression. For snRNA‐seq, libraries were generated from pooled hippocampal tissues (three rats per experimental group). For A‐F, integrated analysis was conducted using data derived from these libraries. All high‐quality nuclei from every experimental group (12 rats total) were aggregated for combined processing, including clustering, cell type annotation, and cell‐cell communication inference. For G and H, representative visualizations from the snRNA‐seq data are shown. All statistical comparisons are based on single‐nucleus level data. (A) Cell‐cell communication networks in the rat hippocampus. Cell types included astrocytes (AST), oligodendrocytes (OLs), oligodendrocyte progenitor cells (OPCs), mural cells (MCs), ependymal cells (Epen), neuronal cells (NCs), endothelial cells (ECs), and microglia (MG). Left: Number of interactions. Node size represents cell numbers, and edge thickness represents ligand‐receptor pair numbers. Right: Interaction strength and weights. Node size represents cell numbers, and edge thickness represents ligand‐receptor pair strength and weights. (B) OPC‐ and oligodendrocyte‐specific communication patterns. Left: Number of interactions between OPCs and other hippocampal cell types. Right: Number of interactions between oligodendrocytes and other hippocampal cell types. (C) Dot plot displaying neuronal subgroup marker gene expression. Cell types included GABAergic neurons (GABA‐Ns), granule cells (GCs), neuronal progenitor cells (NPCs), CA1, CA2, and CA3. Dot size represents the percentage of cells expressing the gene, and dot color represents the average expression level in expressing cells (purple: high). (D) UMAP visualization of neuronal subclusters. Dot colors represented different subgroups. (E) Cell‐cell communication networks between OPCs and neuronal cell subtypes in the hippocampus. Left: Number of interactions among cell types. Right: Interaction strength and weights. (F) OPC‐specific communication patterns. Left: Number of interactions. Right: Interaction strength and weights. (G) Dot plot displaying differentially expressed genes in NPCs of WT and *Crp* KO rats with or without PD. Dot size represented the percentage of cells expressing the gene. Dot color indicated the average expression level in expressing cells (purple: high). (H) mRNA expression levels of selected genes in NPCs, derived from snRNA‐seq data. Statistical significance was determined by the Wilcoxon rank‐sum test (^*^ *p* < 0.05, ^**^ *p* < 0.01, ^***^ *p* < 0.001, ^****^ *p* < 0.0001). (I) Representative IHC staining images of NeuroD1^+^ (white) cells in the DG area of WT and *Crp* KO rats with or without PD. DAPI (blue) marks the nuclei. The blue dashed line indicates ROI. (J) Statistical analysis of NeuroD1^+^ cells across groups. (K) Representative IHC staining images of NeuroD1^+^ (white) cells in the DG area of AAV‐injected WT and *Crp* KO rats with or without PD. DAPI (blue) marks the nuclei. The red dashed line indicates ROI. (L) Statistical analysis of NeuroD1^+^ cells across groups. Results are presented as mean ± SEM (*n* = 4 rats per group for J and L). Statistical significance (^*^ *p* < 0.05, ^**^ *p* < 0.01) was assessed with one‐way ANOVA for J and L.

In the final phase of this study, we performed an analysis of OPCs' interactions with other hippocampal cell populations. Ligand‐receptor interaction analysis revealed communication between OPCs and other cell types, including neuronal cells, astrocytes, ependymal cells, oligodendrocytes, and endothelial cells (Figure 6A,B). Notably, OPCs exhibited substantially more interaction with neuronal cells than with other cell populations. Besides, oligodendrocytes also communicated with neuronal cells, but the number of these interactions was lower than that between OPCs and neuronal cells. Based on our previous findings that PD impairs neurogenesis, we focused subsequent analyses on OPC‐mediated regulatory effects on neuronal cells.

Then we classified hippocampal neuronal cells into seven subtypes, with subtype‐specific mRNA expression patterns in the dot plot (Figure 6C,D). Ligand‐receptor interaction analysis revealed that OPCs could communicate with all neuronal subtypes (Figure 6E,F). Based on these interactions, we analyzed the effects of CRP and PD on gene expression across neuronal subtypes. Notably, in neural progenitor cells (NPCs) of WT rats, PD significantly downregulated 19 mRNAs related to neuronal development, proliferation, and differentiation, including *Neurod1*, *Bhlhe22*, *Aplp1*, and *Camk2b* (Figure 6G,H; Figure S7). *Crp* KO effectively reversed these PD‐induced changes, again demonstrating that CRP deficiency maintains hippocampal neurogenesis during PD. Additionally, while PD elevated expression of the injury‐response gene *Nrg1*, this effect was prevented in *Crp* KO rats (Figure S7), suggesting reduced NPC damage when CRP signaling is blocked.

Finally, IHC analysis revealed that neither PD induction nor Crp KO significantly altered the proportion of Ki67^+^SOX2^+^ cells among SOX2^+^ cells or the percentage of Ki67^+^ cells in the DG (Figure S8), collectively suggesting that NPC proliferation was not significantly affected. We therefore investigated the differentiation stage, focusing on the effect of the CRP‐BMP4 axis on NeuroD1, a transcription factor essential for neuronal differentiation. Consistent with the mRNA changes (Figure 6H), PD reduced NeuroD1^+^ cell numbers by approximately threefold in the DG area of WT rats, while this reduction was prevented by *Crp* KO (Figure 6I,J). However, when *Bmp4* was overexpressed via AAV in PD rats, reduced NeuroD1^+^ cell numbers in the DG area were observed even in *Crp* KO rats (Figure 6K,L). Therefore, CRP regulates NPC differentiation primarily through BMP4‐dependent mechanisms.

**FIGURE 7:**
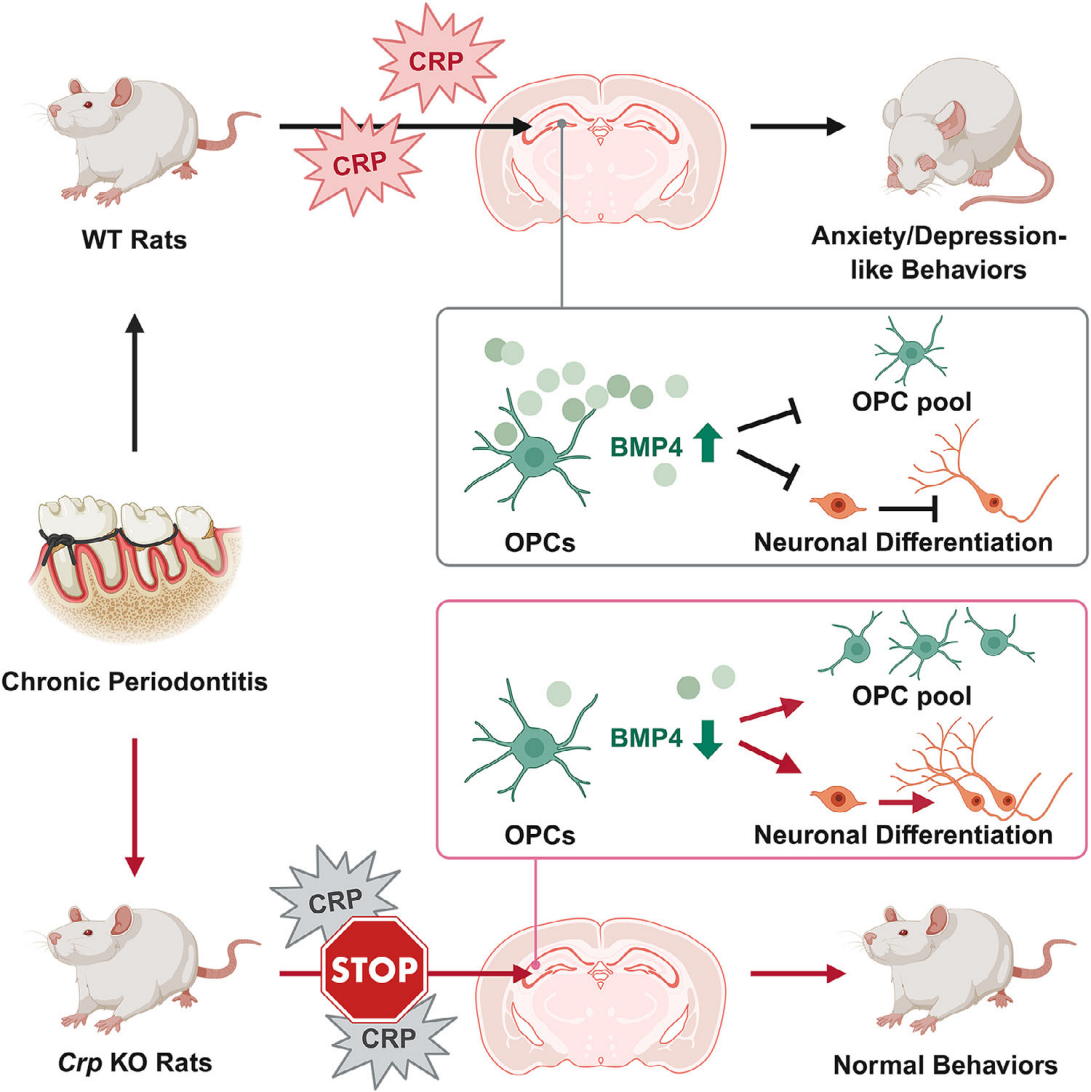
Schematic diagram illustrating the involvement of the CRP‐BMP4 axis in hippocampal impairment and behavioral abnormalities in a rat model of chronic periodontitis.

Collectively, silk ligature‐induced PD elevated hippocampal CRP, which impaired neurogenesis in the DG and induced anxiety/depression‐like behaviors in rats. *Crp* KO alleviated these pathological changes primarily through BMP4‐dependent mechanisms. We identified OPCs as the primary source of BMP4. CRP deficiency inhibited BMP4 expression in OPCs and was associated with alterations in their adaptive capacity and function. Critically, the resulting reduction in BMP4 levels restored PD‐impaired neuronal differentiation of NPCs (Figure 7).

## 3 Discussion

Our study demonstrates that silk ligature‐induced PD contributes to anxiety/depression‐like behaviors and hippocampal neurogenesis impairment through the CRP‐BMP4 axis. Specifically, circulating CRP may reach the hippocampus and regulate BMP4 production in OPCs. CRP deficiency reduces BMP4 in OPCs, thereby preventing PD‐induced OPC loss and rescuing neuronal differentiation potential.

Previous studies showed inconsistent findings on the association between periodontal health and mental disorders such as anxiety and depression [24, 25, 26], likely due to confounding variables (demographics, comorbidities), measurement biases (diagnostic criteria), and study design variations. To specifically examine the relationship of PD and mental disorders, we used an optimized model with figure‐eight silk ligatures around both first and second molars of rats. This approach enhanced plaque accumulation and ligature stability compared to the conventional single‐tooth method. After 4 weeks, ligatured rats showed significant alveolar bone resorption, periodontal inflammation, and anxiety/depression‐like changes (reduced exploration, impaired neurogenesis) resembling rats with chronic stress, but without exacerbating stress‐induced behavior and neurogenesis changes. Notably, while studies employing long‐term oral gavage of periodontal pathogens (2–3 months) reported elevated pro‐inflammatory cytokines *Il‐1β* and *Tnf‐α* in the frontal cortex [27, 28], our 4‐week ligature model did not induce significant increases in these cytokines in the dorsal hippocampus. This finding is consistent with a previous ligature‐induced PD model, which also observed no significant upregulation of *Il‐1β* or *Tnf‐α* in the same region [29]. We propose that this discrepancy can be attributed to several factors. First, the cortex and hippocampus may differ in their susceptibility to PD‐associated stimuli, even though both are involved in emotional regulation [30, 31]. Second, direct oral administration of pathogens may facilitate their access to CNS and trigger a more pronounced neuroinflammatory response. Third, a long exposure duration may be necessary to initiate a classic pro‐inflammatory cytokine surge. Together, these points indicate that the PD‐related CNS impairment likely involves complex, region‑specific mechanisms beyond neuroinflammation.

Building on these findings, we aimed to identify the mediators linking 4‐week silk ligature‐induced PD to impaired neurogenesis in the hippocampus. Using antibody arrays, CRP emerged as a key candidate. This evolutionarily conserved acute‐phase protein serves as a shared risk marker for both PD [32] and mental disorders [33, 34]. CRP is primarily synthesized in the liver and released into the circulation in pentameric form [34], which can undergo a conformational change to the pro‐inflammatory monomeric CRP(mCRP) at sites of tissue injury, a process known to activate complement pathways [35]. To investigate the origin of hippocampal CRP elevation in our ligature‐induced PD model, we first evaluated the possibility of local synthesis. Previous work has shown that *CRP* mRNA is present at very low levels in the normal human brain but is upregulated in the hippocampus of Alzheimer's patients, where increased mRNA levels lead to a corresponding increase in CRP protein [36]. In our study, despite barely detectable *Crp* mRNA levels in the rat hippocampus that showed no change with PD, CRP protein levels increased significantly, a rise that paralleled elevations in serum and periodontal tissues. Therefore, although the possibility of post‐transcriptional regulation enhancing local CRP synthesis cannot be entirely excluded, we propose that the PD‐induced increase in hippocampal CRP primarily originates from peripheral sources.

We next explored how PD promotes CRP entry into the hippocampus. Since the BBB serves as a major gateway for circulating proteins, we evaluated its integrity by examining the expressions of ZO‐1 and CAV‐1. Our results showed that PD did not significantly influence the levels of these structural proteins, suggesting that BBB disruption mediated through changes in ZO‐1 or CAV‐1 expression is not a primary mechanism. However, more subtle alterations in BBB function or alternative transport routes cannot be excluded. For instance, CRP may enter tissues via matrix sieving‑enforced retrograde transcytosis [37], though whether this occurs at the BBB remains unclear. Another possible explanation involves the conformational change of CRP to its monomeric form (mCRP), which has been shown to increase vascular permeability by destabilizing cell–cell junctions [38]. In summary, the pathway through which PD elevates hippocampal CRP remains undefined, necessitating further investigation.

Having established the peripheral origin and hippocampal presence of CRP, we then evaluated its functional role. In *Crp* KO rats, although periodontal tissue damage occurred upon PD induction, hippocampal neurogenesis was significantly less susceptible to PD‐induced impairment. These data indicate that CRP functions as a risk factor linking PD to hippocampal impairment, and its downregulation may represent a potential therapeutic strategy. Notably, although CRP deficiency prevented anxiety/depression‐like behaviors, neurogenesis remained impaired under combined chronic stress and PD conditions, indicating the limits of this protective effect.

We further identified CRP as an upstream regulator of BMP4, a member of the TGF‐β superfamily involved in embryonic development [39], skeletal formation [40], and CNS disorders [41, 42]. Previous studies have established that BMP4 promotes astrogliogenesis [43, 44] while inhibiting neuronal differentiation [41], and can activate astrocytes and oligodendrocytes, as well as upregulate pro‐inflammatory factors [45, 46]. Consistent with this, our study showed that AAV‐mediated *Bmp4* overexpression increased pro‐inflammatory factors, increased or activated astrocytes and microglia, and decreased the number of newborn neurons, whereas *Crp* KO attenuated these changes. However, in our PD model, we observed elevated BMP4 alongside impaired neurogenesis, yet without concurrent significant changes in astrocyte or microglia number or morphology. This discrepancy could be explained by considering two aspects. First, the effects of BMP4 are potentially concentration‐dependent [46]; the levels in our model may have been sufficient to impair neurogenesis without reaching the threshold required to alter astrocytes and microglia. Second, the time course and species‐specific context are likely crucial. Our 4‐week ligature model in rats may capture an earlier pathological stage, as significant co‐activation of both astrocytes and microglia in rats is reported to require a longer duration (8 weeks) [47], whereas mice show responses within 4–6 weeks [14, 29]. Furthermore, the nature of the stimulation is also important: direct systemic stimuli, such as intraperitoneal injection of Pg‐LPS, can induce rapid activation of both astrocytes and microglia within days, [48] whereas oral or ligature‐based models typically lead to a more gradual central response [12].

Therefore, the PD‐induced CRP‐BMP4 axis likely influences the CNS in a concentration‐ and time‐dependent manner. Unraveling the dynamics of this spatiotemporal cascade, including why changes in neurogenesis precede those in astrocytes and microglia and what other factors may be involved, represents an important direction for future research.

Next, to elucidate the role of the CRP‐BMP4 axis in the silk ligature‐induced hippocampal impairments, we performed snRNA‐seq and cell experiments, identifying OPCs (also called NG2 glia) as the primary source of *Bmp4*. As the most abundant resident progenitor population in the brain, OPCs normally differentiate into oligodendrocytes to support myelination and exhibit rapid injury responsiveness [20, 49]. Interestingly, hippocampal astrocytes in adult rats showed nearly undetectable *Bmp4* expression, whereas those in neonatal rats exhibited measurable *Bmp4* levels, though significantly lower than those in OPCs. This pattern suggests an age‐dependent decrease in astrocytic *Bmp4* expression, whereas OPCs maintain relatively stable levels of *Bmp4* expression throughout development.

Further analysis identified OPCs_4, an OPC subpopulation characterized by high expression of the intermediate differentiation markers *Enpp6* and *Chn2*, as the predominant *Bmp4*‐expressing cluster. Considering that *Enpp6* and *Chn2* expression peaks during oligodendroglial lineage progression but declines with maturation [50, 51], and given our snRNA‐seq data showing low *Bmp4* in mature oligodendrocytes, the findings collectively suggest a potential inverse correlation between *Bmp4* levels and oligodendrocyte maturation. This concept found support in subsequent experiments: AAV‐mediated *Bmp4* overexpression reduced both OPC numbers and MBP levels, consistent with known inhibitory effects of BMP signaling on remyelination [52]. We also found that *Bmp4* overexpression induces complex protrusions in some hippocampal OPCs, a pathological feature potentially associated with neuron impairment and disrupted synaptic regulation [53, 54]. Therefore, *Bmp4* overexpression not only inhibits oligodendrocyte maturation but may also impair neuronal function.

We then sought to understand this process in our PD model. Results showed that the mild elevation of *Bmp4* induced by PD alone was sufficient to reduce the OPC pool but did not affect myelination. Furthermore, *Bmp4* overexpression not only further decreased OPC numbers but also disrupted myelination. These findings demonstrate that myelination is more resistant to BMP4 signaling than is the maintenance of OPC numbers, as it withstands BMP4‐mediated OPC loss and is only disrupted when BMP4 levels exceed a critical threshold. Based on the characteristics of our 4‐week ligature model that may capture early hippocampal changes induced by PD, future studies examining different time points would help determine whether PD disrupts myelination and how such impairments progress across various pathological stages.

We next investigated the role of CRP in OPC homeostasis. At baseline, *Crp* KO rats maintained a smaller OPC pool compared to WT rats. The OPCs in *Crp* KO rats exhibited a less differentiated morphology, a migratory phenotype associated with damage repair and potential involvement in neuronal regeneration [55]. Despite these differences, MBP levels were not reduced, showing that the smaller OPC population in *Crp* KO rats remains functionally sufficient to maintain myelination. When challenged with PD, the oligodendroglial lineage in *Crp* KO rats demonstrated a capacity to expand the OPC pool and shift it toward a more mature state, which may represent a compensatory mechanism to safeguard myelination. In addition, an important finding emerged from *Bmp4* overexpression experiments: the reduction in OPC numbers confirmed that the CRP‐BMP4 pathway regulates OPC population size, while the preservation of myelination suggested that *Crp* KO may enhance the functional capability of the remaining OPCs. Collectively, the less differentiated, migratory OPC phenotype at baseline in *Crp* KO rats may reflect a primed state that, under pathological challenge, enables a stronger and more adaptive response, ultimately enhancing the hippocampal capacity to maintain myelination. This shift from a quantity‐dependent to a quality‐driven process could represent one explanation for how *Crp* KO confers protection without increasing baseline OPC numbers.

In summary, although the CRP‐BMP4 axis mediates PD‐induced OPC loss, it cannot account for the preserved myelination in *Crp* KO rats. This discrepancy nevertheless points to the existence of BMP4‐independent mechanisms that enhance OPC function, warranting further investigation.

Finally, cell interaction analysis suggested that OPC communication with other cell types may contribute to the PD‐induced hippocampal impairment. Prior studies have established that OPCs regulate CNS homeostasis through multiple pathways, including astrocyte maturation and proliferation [56] (similar to our observation of increased astrocytes upon *Bmp4* overexpression), glutamatergic signaling in pyramidal neurons, and neuronal electrical activity [53]. Here, our work identifies a mechanism in which CRP deficiency preserves neurogenesis under PD conditions by suppressing *Bmp4* in OPCs. Specifically, PD disrupted the expression of neuronal differentiation‐related genes, an effect reversed by *Crp* KO via *Bmp4* suppression. A clear example is the rescue by *Crp* KO of the PD‐induced decrease in the expression and translation of NeuroD1, a transcription factor essential for neuronal differentiation [57]. Although BMP4‐mediated suppression of NeuroD1 has been previously reported [58], our findings significantly advance the understanding of this regulatory mechanism by identifying CRP as an upstream regulator of the BMP4‐dependent NeuroD1 suppression and demonstrating that CRP deficiency attenuates PD‐induced impairment of neuronal differentiation through this pathway.

In conclusion, our findings demonstrate a pathway through which PD may contribute to hippocampal impairment. We identify the CRP‐BMP4 axis as a key mediator in this process, whereby peripherally‐derived CRP upregulates BMP4 primarily in OPCs, subsequently inhibiting neuronal differentiation. CRP deficiency not only prevents the neurogenesis impairment by suppressing BMP4 expression but may also enhance the adaptive capacity of OPCs, thereby preserving myelination function even under BMP4 overexpression. These findings provide novel insights into how oral disease may lead to CNS dysfunction and suggest potential therapeutic strategies for periodontal disease‐associated neurological complications.

However, several limitations of this study should be considered. First, the pathway through which peripheral CRP enters the brain remains elusive, and clarifying this process is essential for understanding its role in CNS disorders. Second, the mechanisms underlying OPC‐neuronal interactions that contribute to impaired neurogenesis are not fully understood. Future analysis of our snRNA‐seq data could reveal additional intercellular signaling pathways that act either in concert with or independently of the CRP‐BMP4 axis. Third, the impact of PD on the CNS may exhibit spatiotemporal dynamics, with different regions and cell types affected over time. As our model may represent an early phase, future work should incorporate assessments across multiple time points and brain regions to fully elucidate the role of the CRP‐BMP4 axis throughout PD progression. Addressing these aspects will strengthen the translational relevance of our findings and provide deeper insights into the oral‐brain axis.

## 4 Experimental Section

### 4.1 Animals

The Animal Ethics Committee of Chongqing Medical University approved the experiments (No. 2022[063]). Male wild‐type (WT) Sprague‐Dawley (SD) rats, aged 6–8 weeks, were obtained from Chongqing Medical University. *Crp* knockout (KO) rats on an SD background, generated via TALEN technology, were kindly provided by Professor Gangyi Yang from the Second Affiliated Hospital of Chongqing Medical University. Each cage housed two to four rats and was maintained in a pathogen‐free facility with a constant temperature and humidity. They were provided with clean water and food. According to the experimental design, 6–12 rats were divided into each group. Prior to modeling, the rats underwent 1–2 weeks of adaptive feeding.

### 4.2 Chronic Stress (Stress) Model

Empty mineral water bottles were used to create restraining devices intended to limit the movements of rats, preventing them from standing, walking, or turning around. Care is taken to ensure that the rats can breathe comfortably despite these constraints. The rats are continuously restrained for 4–7 h during any given period between 8 am and 7 pm each day, during which time they are deprived of both water and food. Following 4 weeks of restraint, subsequent experiments and testing were performed.

### 4.3 Chronic Periodontitis (PD) Model

After rats had been anesthetized using the inhalation method with isoflurane, the silk ligatures (diameter = 0.2 mm) were placed around the maxillary first and second molars as a figure‐eight. Check the silk ligatures every week to ensure they have not come off. After 4 weeks with the ligatures in place, subsequent experiments and testing were performed.

### 4.4 PD with Stress Model

After 24 h of ligature placement, rats were subjected to restraint stress using empty mineral water bottles. Following 4 weeks of combined ligature maintenance and restraint stress, subsequent experiments and analyses were performed.

### 4.5 Stereotactic Injection

Recombinant adeno‐associated virus (AAV) vectors designed to knock down *Bmp4* (AAV‐*Bmp4*) or to serve as an empty control (AAV‐Empty) in the rat hippocampus were produced by HANBIO Technology (Shanghai, China). The titer was 1×10^12^ (V.g mL^−1^). After being anesthetized with isoflurane, 7‐ to 8‐week‐old rats were fixed in a stereotactic apparatus. Erythromycin ointment was applied to both eyes to prevent keratitis. A burr hole was then drilled at the predetermined injection site. Subsequently, a microsyringe needle was positioned at the following coordinates relative to Bregma: Anterior‐Posterior (AP) −3.8 mm; Medial‐Lateral (ML) ±2.2 mm; Dorsal‐Ventral (DV) −3.0 mm from the skull surface. A volume of 1 µL of AAV was injected bilaterally at a rate of 0.1 µL min^−1^. The needle was left in place for 10 min before being slowly withdrawn. The incision was sutured, and the rats were kept warm until fully recovered from anesthesia. 3 weeks after the injection, PD models were induced using silk ligatures.

### 4.6 Behavioral Testing

The Open field test (OFT) and the elevated plus maze (EPM) were conducted in a dedicated room maintained at 22–24°C, with a consistent light intensity of 50–100 lux at the center of the OFT arena and the open arms of the EPM. In the OFT, each rat was placed in the center of the square open apparatus and allowed 5 min to explore. 24 h later, the rats were tested on the EPM. The EPM was a plus‐shaped platform elevated above the ground, consisting of open arms and closed arms that intersected in the center. The maze was elevated high enough above the floor to ensure that the rats could not easily jump out. Each rat was positioned in the center of the plus sign and given 5 min to explore. Prior to testing, the rats were allowed to acclimate to the testing environment. Throughout the tests, the room was kept quiet. The apparatus was cleaned with 70% ethanol after each trial. The movements of rats were recorded using a video camera and analyzed with specialized behavior tracking software.

### 4.7 Primary Cell Isolation and Culture

The cerebral cortex and hippocampus were collected from postnatal day 1 (P1) rats. After careful removal of meninges and blood vessels, the tissues were minced into 1‐mm^3^ fragments and digested for 15 min at 37°C with a digestion solution containing 0.25% trypsin (Gibco, 15050065), DNase I (MCE, HY‐108882), and Hanks’ balanced salt solution (HBSS, Beyotime, C0218). The digestion was terminated by adding DMEM/F‐12 (Gibco, C11330500BT) supplemented with 10% fetal bovine serum (FBS). After centrifugation, the pellet was resuspended in culture medium, filtered through a 70‐µm cell strainer to obtain a single‐cell suspension, and plated in poly‐D‐lysine (PDL, Gibco, A3890401)‐coated T75 cell culture flasks. After 7–10 days, the mixed glial cultures were shaken at 200 rpm at 37°C for 1 h. The microglia‐enriched cell suspension was collected and centrifuged. The pellet was resuspended in DMEM/F‐12 medium containing 10% FBS and re‐seeded onto PDL‐coated culture dishes. After microglia collection, the cultures were maintained on an orbital shaker at 200 rpm for 18–20 h to isolate OPCs. The supernatant was sequentially filtered through a 40‐µm cell strainer and centrifuged. The pellet was resuspended in DMEM/F‐12 medium supplemented with N2 (Gibco, 17502048), B27 (Gibco, 17504044), PDGF‐AA (10 ng mL^−1^, PeproTech, 100–13A), bFGF (10 ng mL^−1^, PeproTech, 400–29), and EGF (PeproTech, 400–25). The OPC suspension was then reseeded on PDL‐coated dishes. After OPC collection, the flasks were treated with 0.25% trypsin and incubated at 37°C for 1 min to dissociate the remaining cells. The digestion was terminated by adding DMEM/F‐12 medium supplemented with 10% FBS. After centrifugation, the pellet was resuspended and plated onto PDL‐coated culture dishes for astrocyte culture. All cells were maintained in a humidified atmosphere with 5% CO_2_ at 37°C.

### 4.8 Nissl Staining and Immunohistochemical (IHC) Staining

Paraffin sections of brain and maxilla tissues were prepared separately before staining. The rats were anesthetized with isoflurane and then subjected to cardiac perfusion using pre‐cooled phosphate buffer solution (PBS). After euthanasia, the brain or maxilla tissues were collected and fixed in 4% paraformaldehyde (PFA). Before embedding in paraffin, the maxillae needed to be decalcified in 10% ethylenediaminetetraacetic acid (EDTA). Subsequently, the target region was embedded in paraffin and sectioned to a thickness of 5 µm. Nissl staining was utilized to visualize the arrangement of neurons. Following dewaxing and hydration, the sections were stained with toluidine blue (Servicebio, G1036). The sections were then dehydrated, dried, and finally mounted in neutral resin for preservation. IHC staining was employed to visualize the target proteins. For the IHC procedure, the sections were first dewaxed and hydrated, followed by antigen retrieval using citric buffer (pH 6.0) or Tris‐EDTA solution (pH 9.0) (Servicebio, G1202 and G1218). After incubation with a blocking solution (Beyotime, P0260), the sections were incubated with the primary antibodies at 4°C overnight, and then with the fluorescently labeled secondary antibodies at room temperature for 2 h. Finally, the sections were mounted using an antifade mounting medium containing DAPI (HISTOV, CMT110D). Images were captured using the inverted fluorescence microscope (ZEISS, Axio Observer 7) and the slide scanner (OLYMPUS, VS200). Cell morphology was characterized and analyzed using ImageJ (Fiji, v1.54p). Following image acquisition, individual cells were isolated by applying intensity thresholding and the “Analyze Particles” function. Subsequently, skeletonized images were generated using the “Skeletonize” plugin to extract key morphometric parameters. The primary quantifications included the number of junctions per cell (representing branch points) and the number of endpoint voxels per cell (representing terminal points).

The primary antibodies used were as follows: rabbit anti‐Doublecortin (Abcam, ab207175), mouse anti‐NeuN (abcam, ab104224), rabbit anti‐IL‐1β (Abcam, ab283818), rabbit anti‐TNF‐α (Affinity, AF7014), rabbit anti‐GFAP (Proteintech, 16825‐1‐AP), rat anti‐IBA1 (Abcam, ab283346), rabbit anti‐OLIG2 (Oasis Biofarm, OB‐PRB009), guinea pig anti‐NG2 (Oasis Biofarm, OB‐PGP002), rabbit anti‐MBP (Oasis Biofarm, OB‐PRB130), and rabbit anti‐NeuroD1 (Abcam, ab213725), rabbit anti‐ZO1 (Abcam, ab221547), mouse anti‐Caveolin‐1 (Servicebio, GB15409), rabbit anti‐Ki67 (Servicebio, GB111499), rabbit anti‐SOX2 (Servicebio, GB11249). The fluorescently labeled secondary antibodies used were as follows: goat anti‐mouse IgG H&L (Alexa Fluor 488, Abcam, ab150113), goat anti‐rabbit IgG H&L (Alexa Fluor 647, Abcam, ab150079), goat anti‐rat IgG H&L (FITC, ZSGB‐BIO, ZF‐0315), and goat anti‐guinea pig IgG (Alexa Fluor 647, Oasis Biofarm, G‐GP647).

### 4.9 Immunofluorescence (IF) Staining

The culture medium was removed, and cells were rinsed with pre‐cooled PBS. Cells were then fixed with 4% paraformaldehyde at room temperature for 20 min and blocked with 5% goat serum at room temperature for 1 h. After blocking, cells were incubated overnight at 4°C: rat anti‐IBA1 (Abcam, ab283346), rabbit anti‐OLIG2 (Oasis Biofarm, OB‐PRB009), guinea pig anti‐NG2 (Oasis Biofarm, OB‐PGP002), rabbit anti‐GFAP (Proteintech, 16825‐1‐AP), and rabbit anti‐BMP4 antibody (Abcam, ab39973). Cells were then incubated for 2 h at room temperature with secondary antibodies: goat anti‐rabbit IgG H&L (Alexa Fluor 488, Abcam, ab150077), goat‐anti‐guinea pig IgG (AF647, Oasis Biofarm, G‐GP647). Nuclei were stained with DAPI (HISTOV, CMT110D). Fluorescent images were captured using the inverted fluorescence microscope (ZEISS, Axio Observer 7).

### 4.10 Real‐time Quantitative PCR (Real‐Time qPCR)

After euthanasia, hippocampal tissues from the areas of interest were collected and washed with pre‐cooled PBS. Total RNA from tissues was isolated using an isolation kit (Beyotime, R0027). For primary cells, total RNA was also extracted using the same kit (Beyotime, R0027). Then, the total RNA from tissues and primary cells was reverse transcribed to cDNA with PrimeScript Master Mix (TaKaRa, RR036A). Real‐time qPCR was performed using TB Green Premix Ex Taq II (TaKaRa, RR420B) and primers. The Primers used targeted *Gapdh*, *Il‐1β*, *Tnf‐α*, and *Bmp4*, with their sequences listed in Table S1.

### 4.11 Inflammation Antibody Array

Hippocampal tissues were lysed and analyzed using a rat inflammation antibody array (RayBiotech, AAR‐INF‐3‐2) for inflammatory mediator profiling. Array membranes were blocked at room temperature for 1 h, then incubated with tissue lysates overnight at 4°C. After washing, biotinylated detection antibody cocktail was added and incubated overnight at 4°C, followed by HRP‐streptavidin incubation for 2 h at room temperature. Chemiluminescent signals were developed using immobilon HRP Substrate (Millipore, WBKLS) and imaged with a chemiluminescence system (Bio‐Rad, Chemidoc MP imaging system).

### 4.12 Western Blotting

Hippocampal tissues, gingival tissues, and primary cells were lysed in radioimmunoprecipitation assay (RIPA) buffer (Epizyme, PC104) supplemented with protease and phosphatase inhibitors (Epizyme, GRF101 and GRF102; Beyotime, ST506) for protein extraction. After denaturation in the loading buffer (Epizyme, LT101), 20–60 µg of protein samples were separated by 10% sodium dodecyl sulfate‐polyacrylamide gel electrophoresis (SDS‐PAGE) gels (Epizyme, PG212) and electrotransferred to polyvinylidene difluoride (PVDF) membranes (Millipore, ISEQ00010). Following this, membranes were blocked with non‐fat milk for 1 h at room temperature, then incubated with primary antibodies overnight at 4°C, followed by incubated with horseradish peroxidase (HRP)‐conjugated secondary antibodies for 1 h at room temperature. Finally, the immunoreactive bands were developed using Immobilon Western HRP Substrate (Millipore, WBKLS) and imaged with a chemiluminescence system (Bio‐Rad, Chemidoc MP imaging system).

The primary antibodies used were as follows: rabbit anti‐GAPDH (Proteintech, 10494‐1‐AP), rabbit anti‐CRP (Abcam, ab259862), rabbit anti‐SMAD1/5/9 (Abcam, ab300164), rabbit anti‐phospho‐SMAD1/5/9 (Cell Signaling Technology, 13820), rabbit anti‐BMP4 (Abcam, ab39973) and rabbit anti‐MBP (Oasis Biofarm, OB‐PRB130). The HRP‐conjugated secondary antibodies used was goat anti‐rabbit IgG (ZSGB‐BIO, ZB2301).

### 4.13 Enzyme‐Linked Immunosorbent Assay (ELISA)

Serum samples were obtained from rat peripheral blood via centrifugation. Following dilution with double‐distilled water, CRP levels were determined using an ELISA kit (Servicebio, GER0007) according to the manufacturer's protocol. Absorbance readings at 450 nm were acquired using a microplate reader, with sample concentrations calculated against the standard curve.

### 4.14 Micro‐CT

Following euthanasia, rat maxillae were dissected and hemisected. Tissues underwent fixation in 4% PFA before micro‐computed tomography scanning (vivaCT40, SCANCO Medical AG). Image reconstruction was performed using the manufacturer's proprietary software.

### 4.15 Statistical Analysis

Statistical analyses were performed using GraphPad Prism (v9.1). Data are presented as mean ± SEM from independent biological replicates, and key experiments were repeated unless otherwise indicated. Comparisons between two groups were performed using a Student's *t*‐test, unless specified. Comparisons among three or more groups were analyzed by one‐way ANOVA followed by appropriate post hoc tests, unless specified. A *p* value < 0.05 was considered statistically significant, with levels denoted as *p < 0.05, **p < 0.01, ***p < 0.001, ****p < 0.0001.

### 4.16 mRNA Sequencing (mRNA‐seq)

#### 4.16.1 RNA Isolation

Hippocampal tissues from WT and *Crp* KO rats (*n* = 3–4 per group) were snap‐frozen in liquid nitrogen and stored at ‐80°C. Total RNA was extracted using TRIzol reagent (Invitrogen, 15596026CN). RNA integrity was verified by agarose gel electrophoresis and purity was confirmed using a NanoPhotometer spectrophotometer (Implen), with all samples meeting the criteria of A260/280>1.8 and A260/230>2.0.

#### 4.16.2 Library Preparation

RNA libraries were prepared using the NEBNext Ultra II RNA Library Prep Kit (New England Biolabs) following the manufacturer's protocol. Briefly, poly(A)^+^ mRNA was fragmented and reverse transcribed using random hexamer primers and M‐MuLV reverse transcriptase. Double‐stranded cDNA was synthesized, and fragments of 250–300 bp were selected using AMPure XP beads (Beckman Coulter, A63882). After end repair, A‐tailing, and adapter ligature, library quality was assessed using Qubit 2.0 Fluorometer (Thermo Fisher Scientific, Q32866) and Agilent 2100 Bioanalyzer (Agilent Technologies), with insert sizes ranging from 420–650 bp. Qualified libraries were sequenced on an Illumina NovaSeq 6000 platform (150 bp paired‐end reads, ≥6 Gb per sample).

#### 4.16.3 Data Processing and Analysis

Raw sequencing data were quality‐controlled using FastQC (v0.11.9) and processed with Trimmomatic (v0.39) to remove adapter sequences and low‐quality bases. For Principal component analysis (PCA), we used the R package stats (version 3.6.0) for analysis. Specifically, we first performed z‐score normalization on the expression profile, and then used the prcomp function for dimensionality reduction analysis to obtain the dimensionality‐reduced matrix. Differential expression analysis was conducted using the R package DESeq2 (Version 1.48.1) with genes considered differentially expressed at |log2 fold change| >0 and *p* value <0.05. Results were visualized using volcano plots with ggplot2.

### 4.17 Single Nucleus RNA Sequencing (snRNA‐seq)

#### 4.17.1 Single Nuclei Isolation

Hippocampal tissues from WT and *Crp* KO rats with or without PD (3 rats per group) were collected and washed with cold PBSE. Nuclei isolation was carried out using GEXSCOPE Nucleus Separation Solution (Singleron Biotechnologies) according to the manufacturer's protocol. Isolated nuclei were resuspended in PBSE at a concentration of 10^6^ nuclei per 400 µL, filtered through a 30 µm cell strainer, and quantified using Trypan blue staining. Nuclei integrity was confirmed by DAPI staining (singlet population).

#### 4.17.2 Library Preparation and Sequencing

The concentration of single nucleus suspension was adjusted to 3–4 × 10^5^ nuclei ml^−1^ in PBS. Libraries were prepared using the GEXSCOPE Single Nucleus RNA‐seq Kit (Singleron Biotechnologies) with microfluidic chip loading per manufacturer's instructions. Sequencing was performed on an Illumina platform (150‐bp paired‐end reads).

#### 4.17.3 Data Processing

Quality control and analysis were conducted using Seurat (v5.0.3). Cells were filtered based on the following criteria: (1) <200 detected genes or top 2% gene counts, (2) top 2% UMI counts, (3) >20% mitochondrial content, and (4) genes detected in <5 cells. After filtering, 93 509 high‐quality cells were retained for downstream analysis, comprising 26 166 from Blank (WT), 22 954 from PD (WT), 22 595 from Blank (*Crp* KO), and 21 794 from PD (*Crp* KO). Dimensionality reduction and clustering were performed using Seurat's standard workflow, with UMAP for 2D visualization.

#### 4.17.4 Analysis

Cluster‐specific differentially expressed genes (DEGs) were identified using Seurat's FindMarkers function with Wilcoxon rank‐sum test (thresholds: expressed in >10% of cluster cells, |log2FC| >0.25, and adjusted *p*<0.05). Cell types were annotated based on canonical markers from the SynEcoSys database (Singleron Biotechnologies). Marker expression patterns were visualized through dot plots and violin plots. Cell‐cell interactions were predicted using the aggregated single‐nucleus data with CellChat (v1.6.1) by analyzing ligand‐receptor pairs.

## Conflicts of Interest

The authors declare no conflict of interest.

## Supporting information




**Supporting File**: advs74852‐sup‐0001‐SuppMat.docx.

## Data Availability

The data that support the findings of this study are available from the corresponding author upon reasonable request.
