# Supplementary material for: CRP Deficiency Rescues Periodontitis‐Induced Hippocampal Neurogenesis Impairment by Suppressing OPC‐Derived BMP4 Signaling in Rats
Source: Adv Sci (Weinh). 2026 Mar 17;13(30):e16199. doi: 10.1002/advs.202516199 (PMC13248755; doi:10.1002/advs.202516199)
Supplement: Supplementary file 1 — Supporting File: advs74852‐sup‐0001‐SuppMat.docx. [file ADVS-13-e16199-s001.docx]

**CRP Deficiency Rescues Periodontitis-Induced Hippocampal Neurogenesis Impairment by Suppressing OPC-Derived BMP4 Signaling in Rats**

Lingjie Li^a, b^*, Ping Deng^a, c^, Siyu Hou^d^, Xianbo Xia^a, c^, Wei Zhao^a, c^, Xingyu Zhu^a, c^, Yang Zhang^a, b^, Chao Wang^b, e^, Ling Xu^a, b^*, Jinlin Song^a, b, c^*

**Affiliations**

^a^ College of Stomatology, Chongqing Medical University, Chongqing, China

^b^ Stomatological hospital of Chongqing Medical University, Chongqing, China

^c^ Chongqing Municipal Key Laboratory for Oral Biomedical Engineering of Higher Education, Chongqing, China

^d^ College of Stomatology, Xi'an Jiaotong University, Xian, China

^e^ School of Biological Science and Medical Engineering, Beihang University, Beijing, China

**Corresponding author**

Dr. Jinlin Song

College of Stomatology, Chongqing Medical University

Chongqing 401147, China.

Email: songjinlin@hospital.cqmu.edu.cn

Dr. Lingjie Li

College of Stomatology, Chongqing Medical University

Chongqing 401147, China.

Email: [501397@hospital.cqmu.edu.cn](mailto:501397@hospital.cqmu.edu.cn)

Dr. Ling Xu

College of Stomatology, Chongqing Medical University

Chongqing 401147, China.

Email: 500203@hospital.cqmu.edu.cn

L.J.L. and P.D. contributed equally as co-first authors. L.J.L. also serves as a co-corresponding author.

**Supplementary Figures**


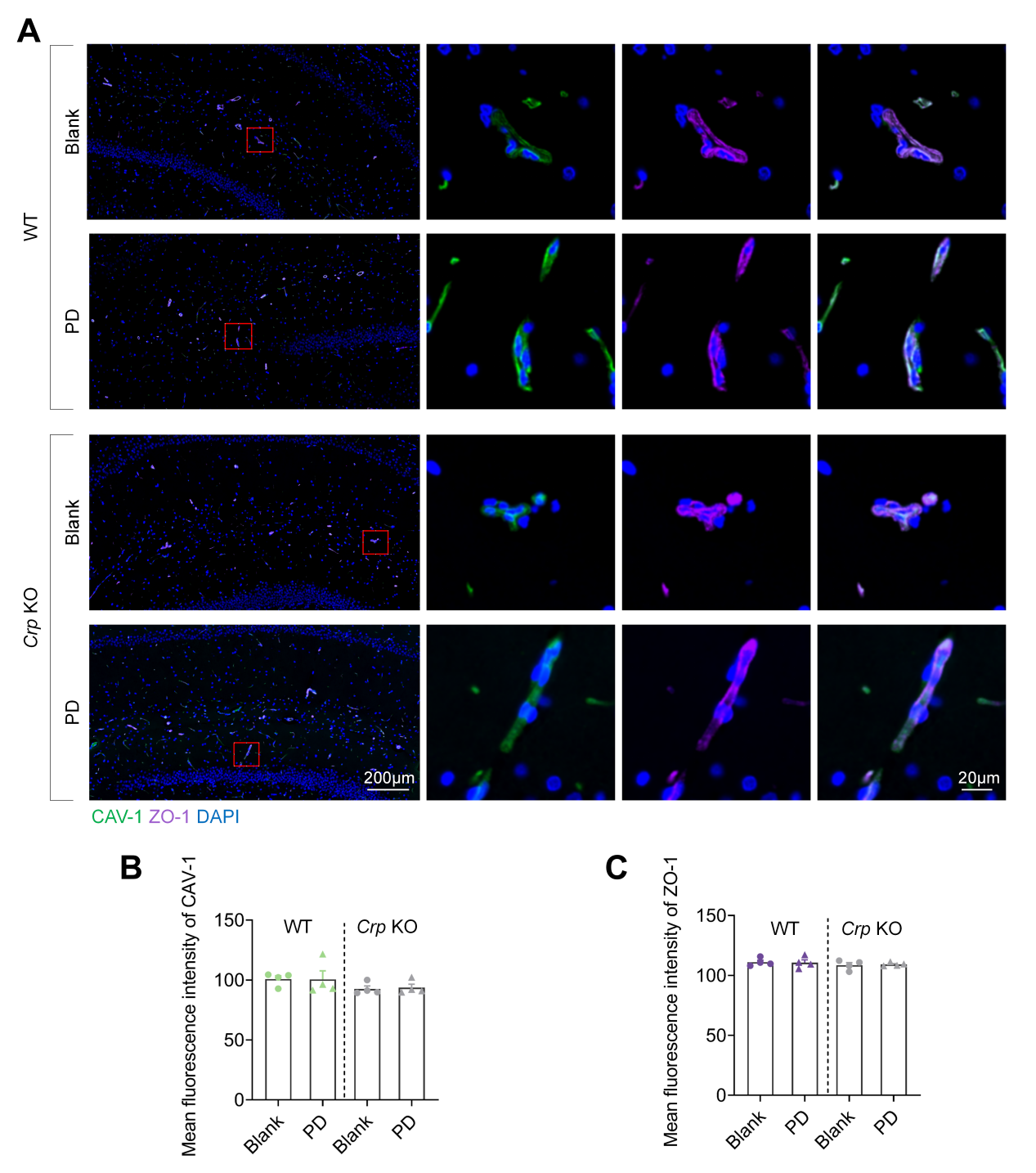


**Figure S1.** Expression of BBB-related proteins CAV-1 and ZO-1 in the hippocampus of WT and *Crp* KO rats with or without PD. A) Representative IHC staining images of CAV-1^+^ (green) and ZO-1^+^ (purple). DAPI (blue) marks the nuclei. Statistical analysis of fluorescence intensity for CAV-1 B) and ZO-1 C). Data are presented as mean ± SEM (n = 4 rats per group). No significant differences were observed.


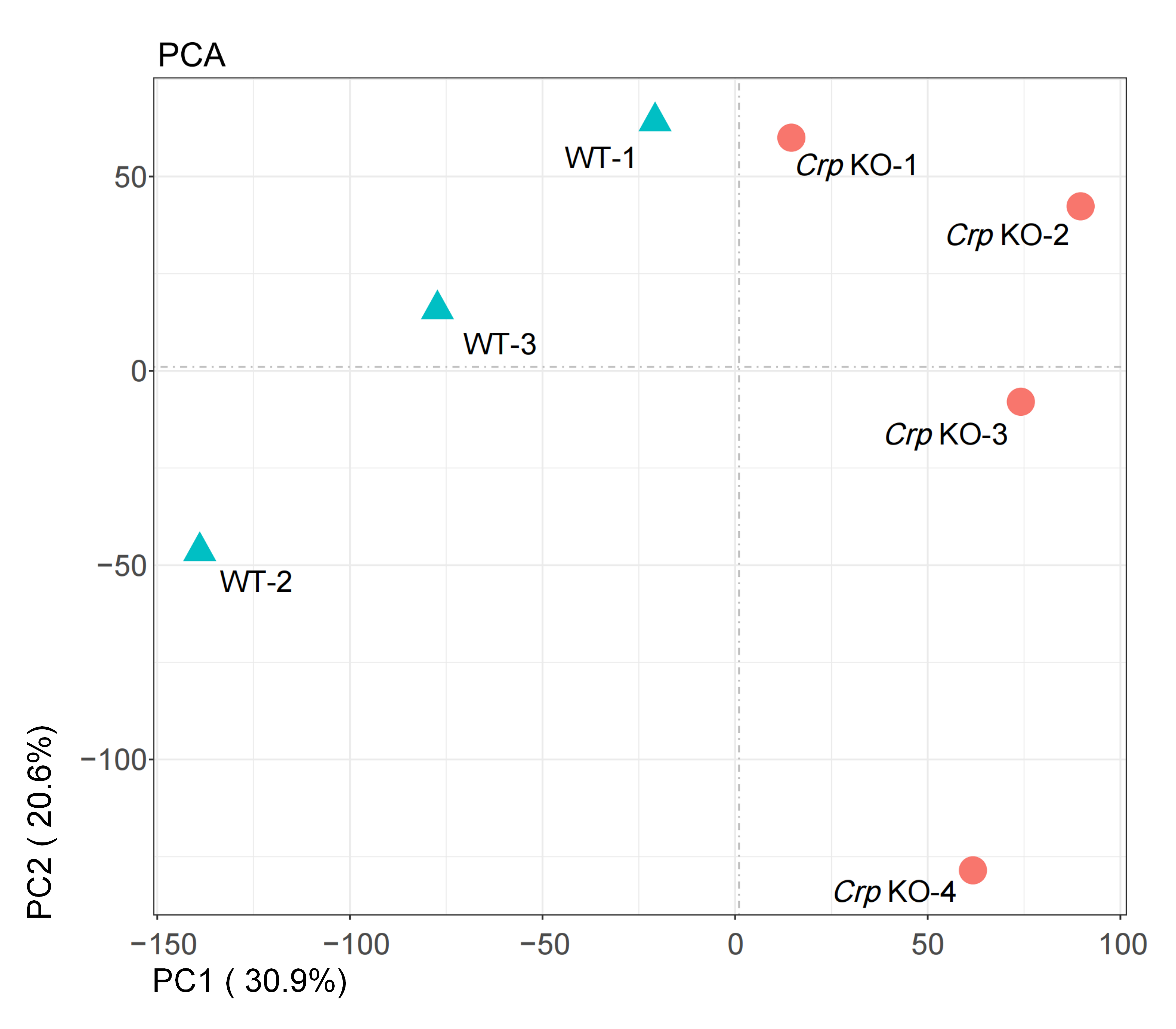


**Figure S2.** Principal component analysis (PCA) of mRNA-seq data obtained from hippocampal tissues of three WT and four *Crp* KO rats.


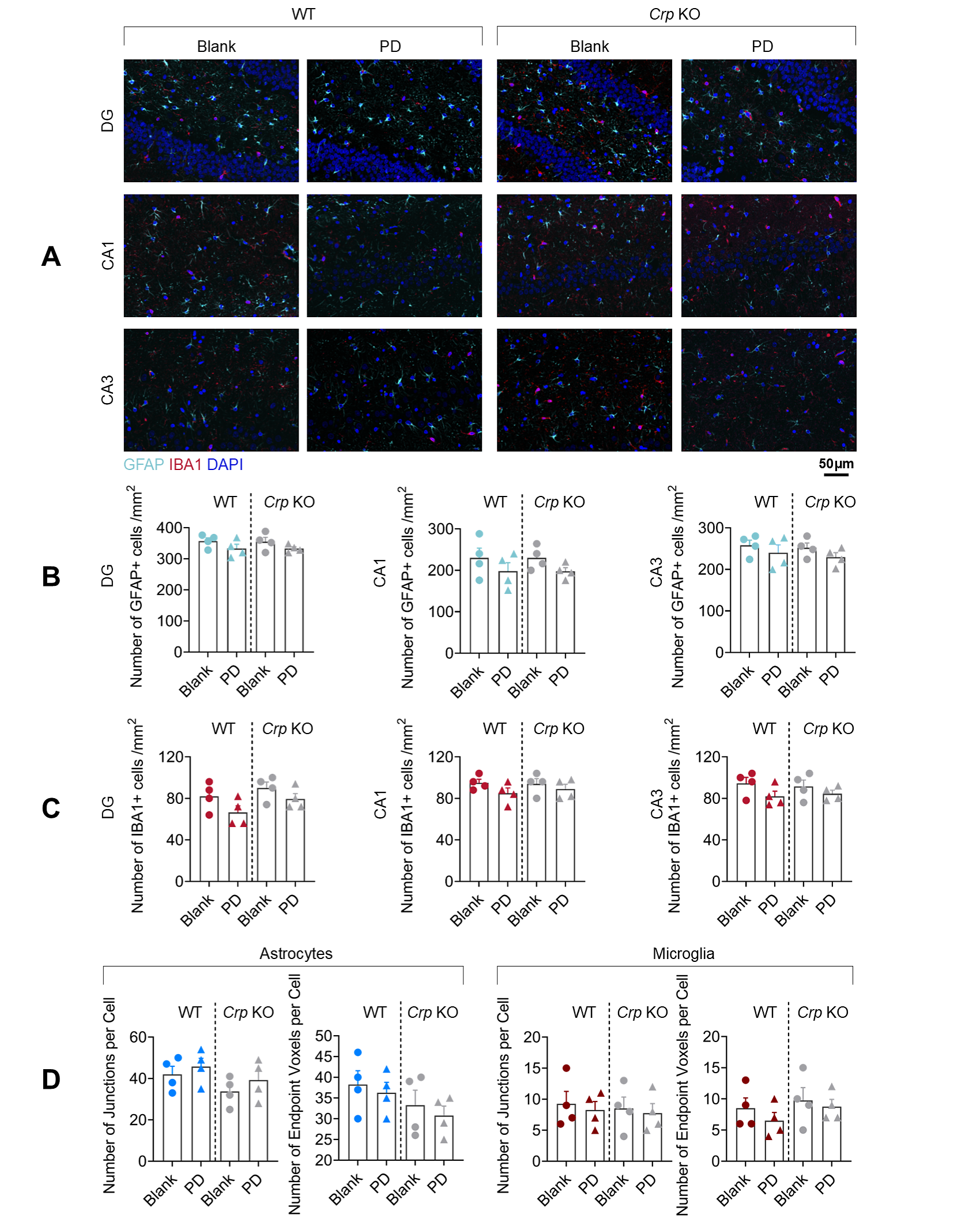


**Figure S3.** Hippocampal astrocyte and microglia number and morphology in rats with or without PD. A) Representative IHC staining images of astrocytes (GFAP^+^, cyan) and microglia (IBA1^+^, red) in the hippocampus of WT and *Crp* KO rats, with or without PD. DAPI (blue) marks the nuclei. Statistical analysis of astrocyte (B) and microglia (C) numbers. D) Morphological analysis of hippocampal astrocytes (left) and microglia (right). Quantification included junction count per cell and endpoint voxels per cell, based on 6 cells per hippocampus for each rat. Data are presented as mean ± SEM (n = 4 rats per group). No significant differences were observed between groups.


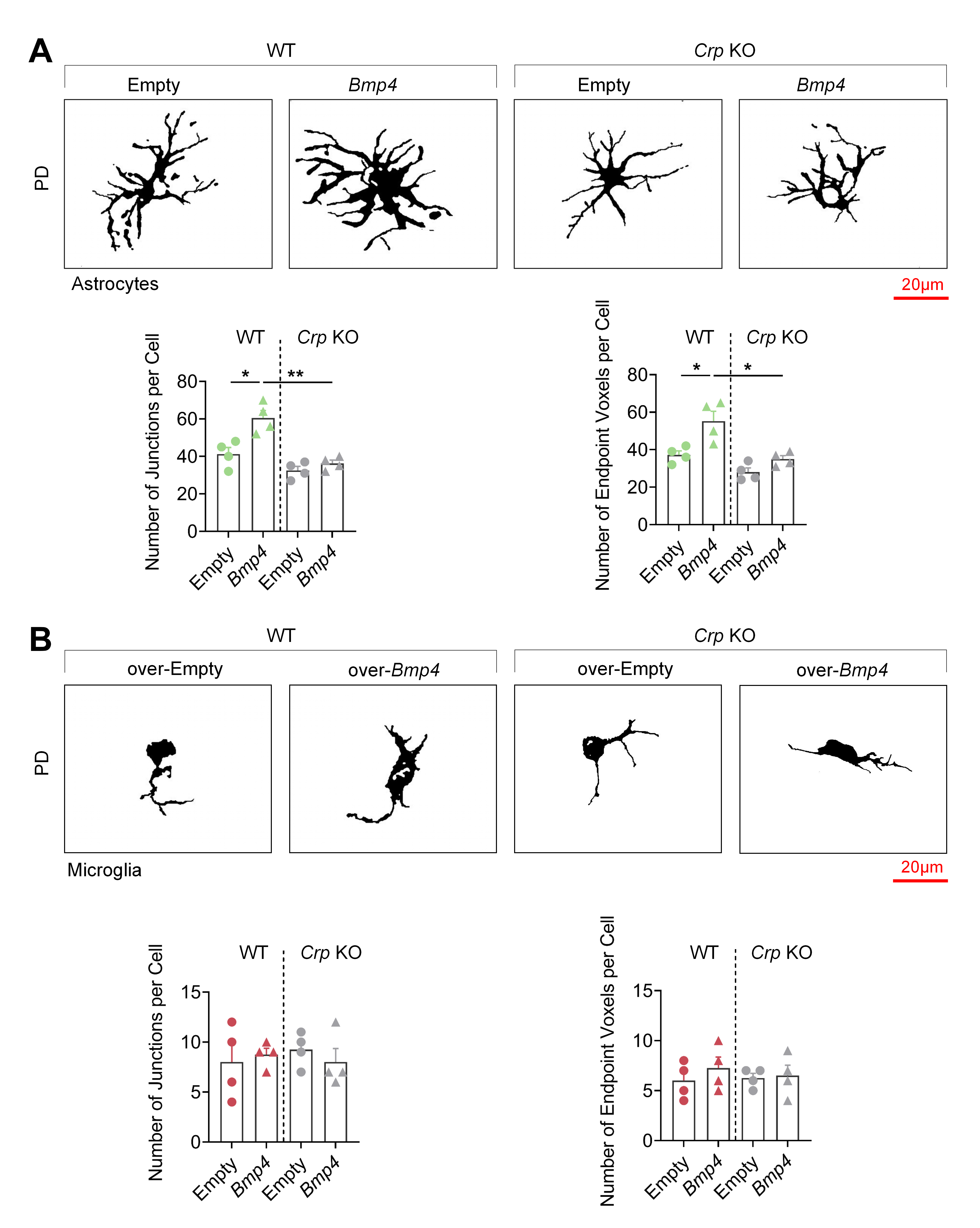


**Figure S4.** Hippocampal astrocyte and microglia morphology in response to PD and *Bmp4* overexpression. Morphological parameters (junctions per cell and endpoint voxels per cell) were quantified from 6 cells per hippocampus for each rat. Data are presented as mean ± SEM (n = 4 rats per group). A) Representative images and statistical analysis of hippocampal astrocytes in WT and *Crp* KO rats after AAV injection (empty vector or *Bmp4*-overexpressing) and subsequent PD induction. (**p* < 0.05, ***p* < 0.01). B) Representative images and statistical analysis of microglia morphology in WT and *Crp* KO rats after AAV injection (empty vector or *Bmp4*-overexpressing) and subsequent PD induction. No significant differences were observed.


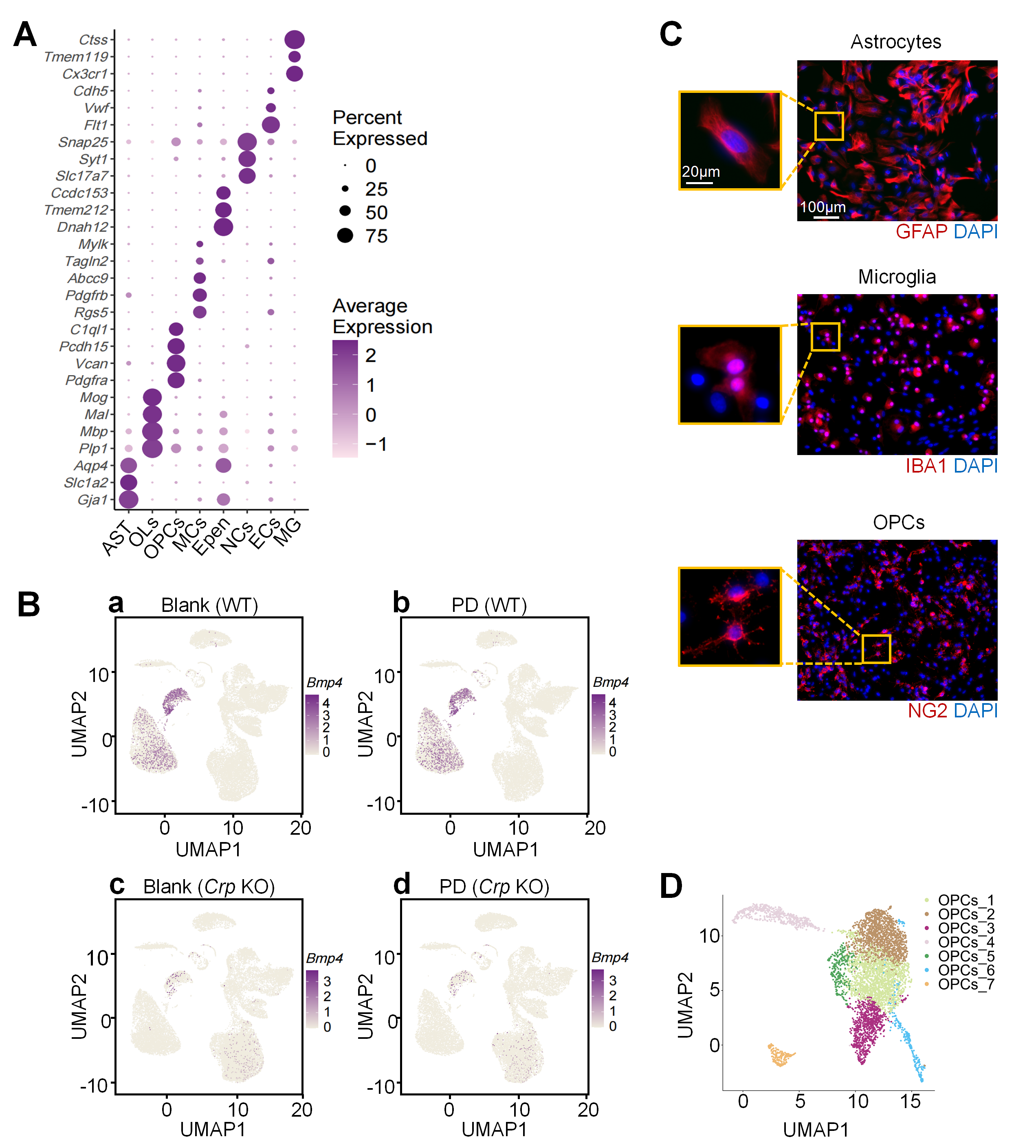


**Figure S5.** snRNA-seq and immunofluorescence characterization of hippocampal cell populations. A) Dot plot of marker gene expression across hippocampal cell subpopulations. Dot size represents the percentage of cells expressing the gene; color indicates the average expression level in expressing cells (purple: high). This analysis is based on snRNA‑seq of hippocampal tissue pooled from 12 rats, with all visualizations presented at the single‑nucleus level. B) snRNA‑seq analysis of *Bmp4* expression in hippocampal cell subpopulations. The sequencing library was prepared by pooling hippocampal tissues from 3 rats per group, and all downstream analyses were performed at the single‑nucleus level. (a–d) UMAP visualizations of hippocampal cell clusters across experimental groups: (a) Blank (WT), (b) PD (WT), (c) Blank (*Crp* KO), and (d) PD (*Crp* KO). Color gradient indicates *Bmp4* expression levels (purple: high; sand: low). C) Representative IF staining images of GFAP^+^ astrocytes, IBA1^+^ microglia, and NG2^+^ OPCs isolated from neonatal WT rat brains. Yellow boxes highlight ROIs, with magnified views shown in the left panels. DAPI (blue) marks the nuclei. D) UMAP plot displaying each OPC subtype, with distinct colors representing different subpopulations. This analysis is based on snRNA‑seq of hippocampal tissue pooled from 12 rats, with all visualizations presented at the single‑nucleus level.


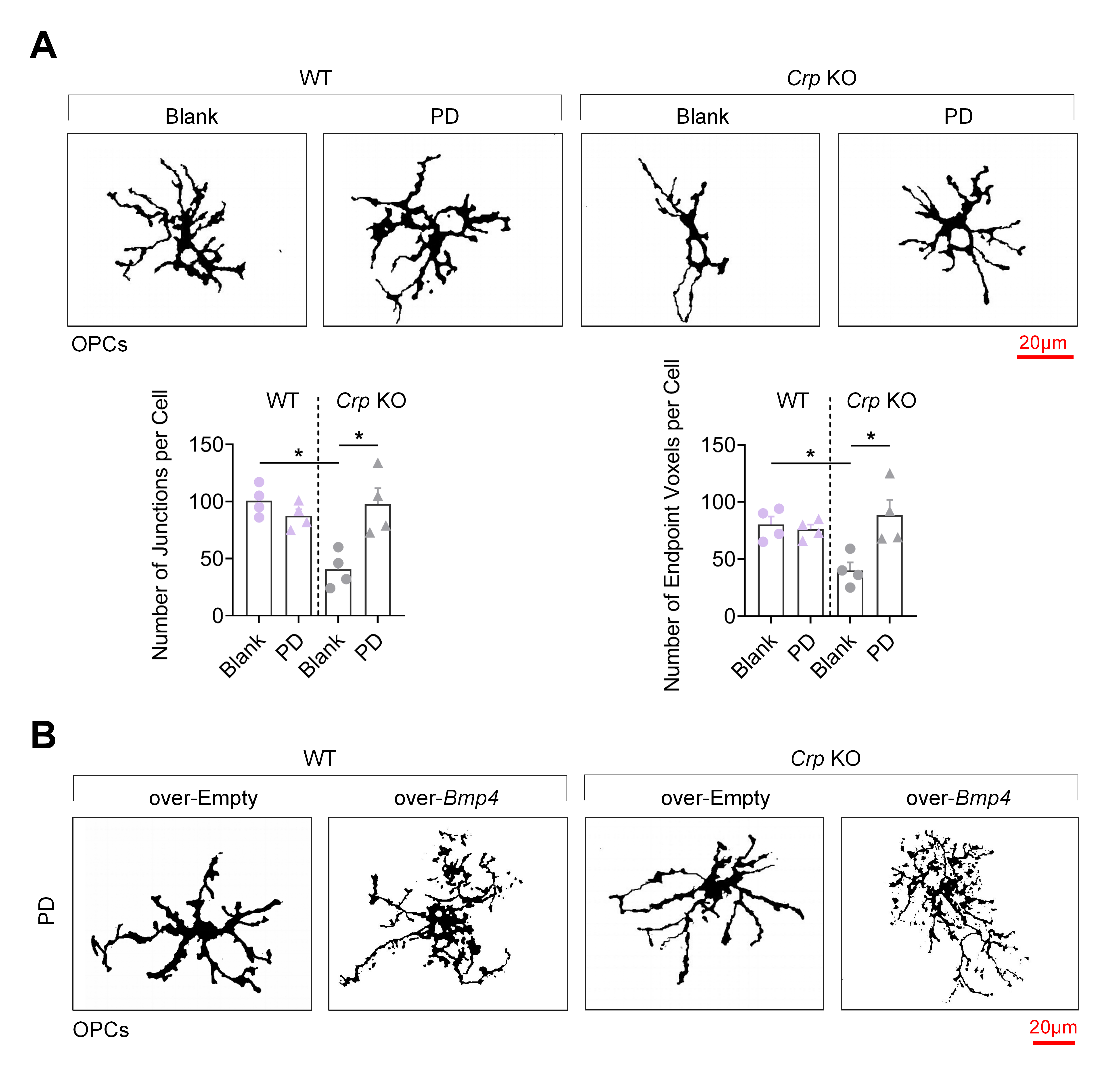


**Figure S6.** Hippocampal OPC morphology in response to PD and *Bmp4* overexpression. A) Representative images and quantifications of hippocampal OPC morphology in WT and *Crp* KO rats with or without PD. Morphological parameters (junctions per cell and endpoint voxels per cell) were quantified from 6 cells per hippocampus for each rat. Group data are presented as mean ± SEM (n = 4 rats per group; **p* < 0.05). B) Representative images of hippocampal OPC morphology in WT and *Crp* KO rats after AAV injection (empty vector or *Bmp4*-overexpressing) and subsequent PD induction.


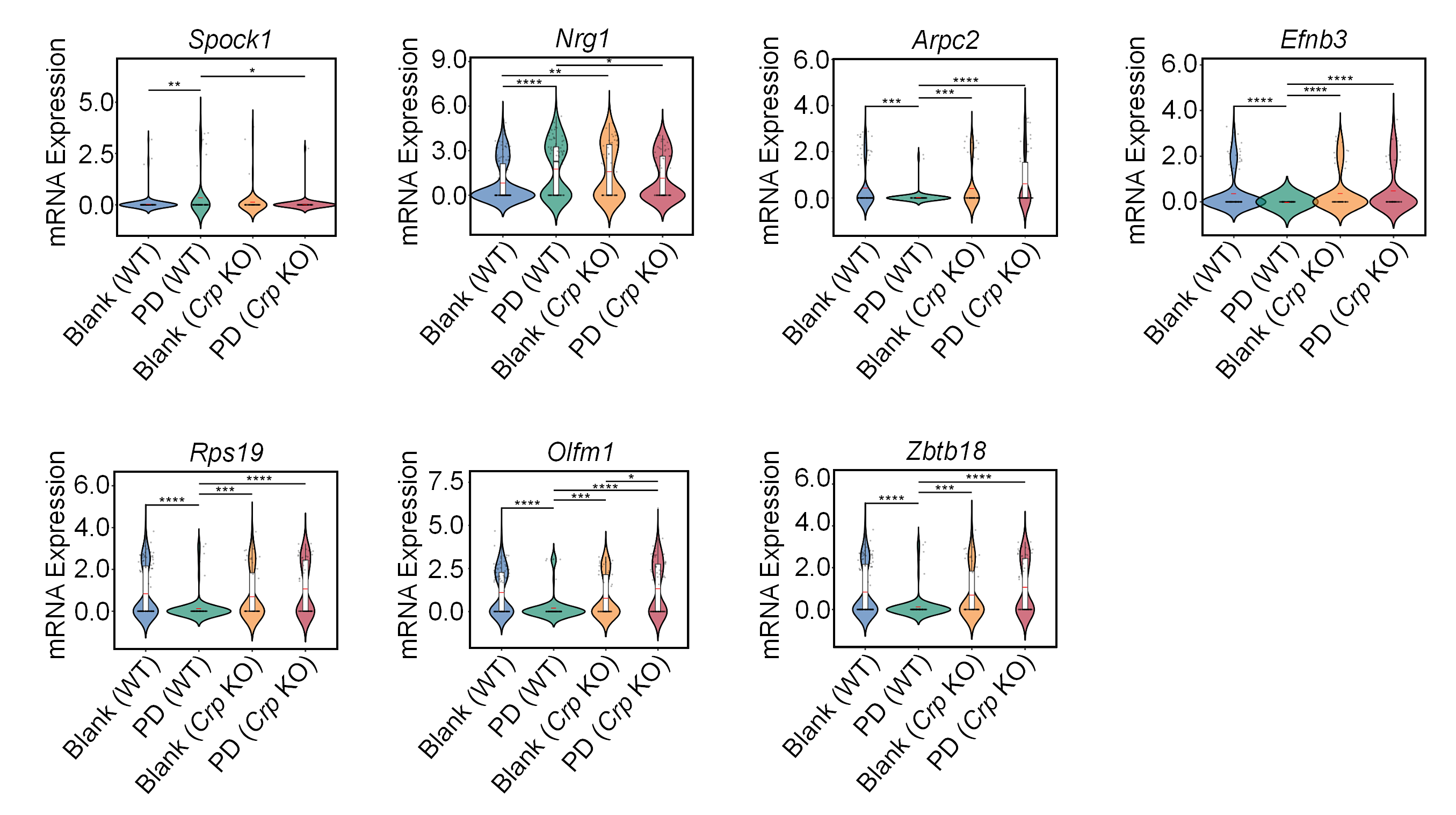


**Figure S7.** mRNA expression levels of selected genes in NPCs, derived from snRNA‑seq data. Hippocampal tissues from 3 rats per experimental group were pooled for library preparation. Statistical significance was determined by the Wilcoxon rank-sum test (**p* < 0.05, ***p* < 0.01, ****p* < 0.001, *****p* < 0.0001).


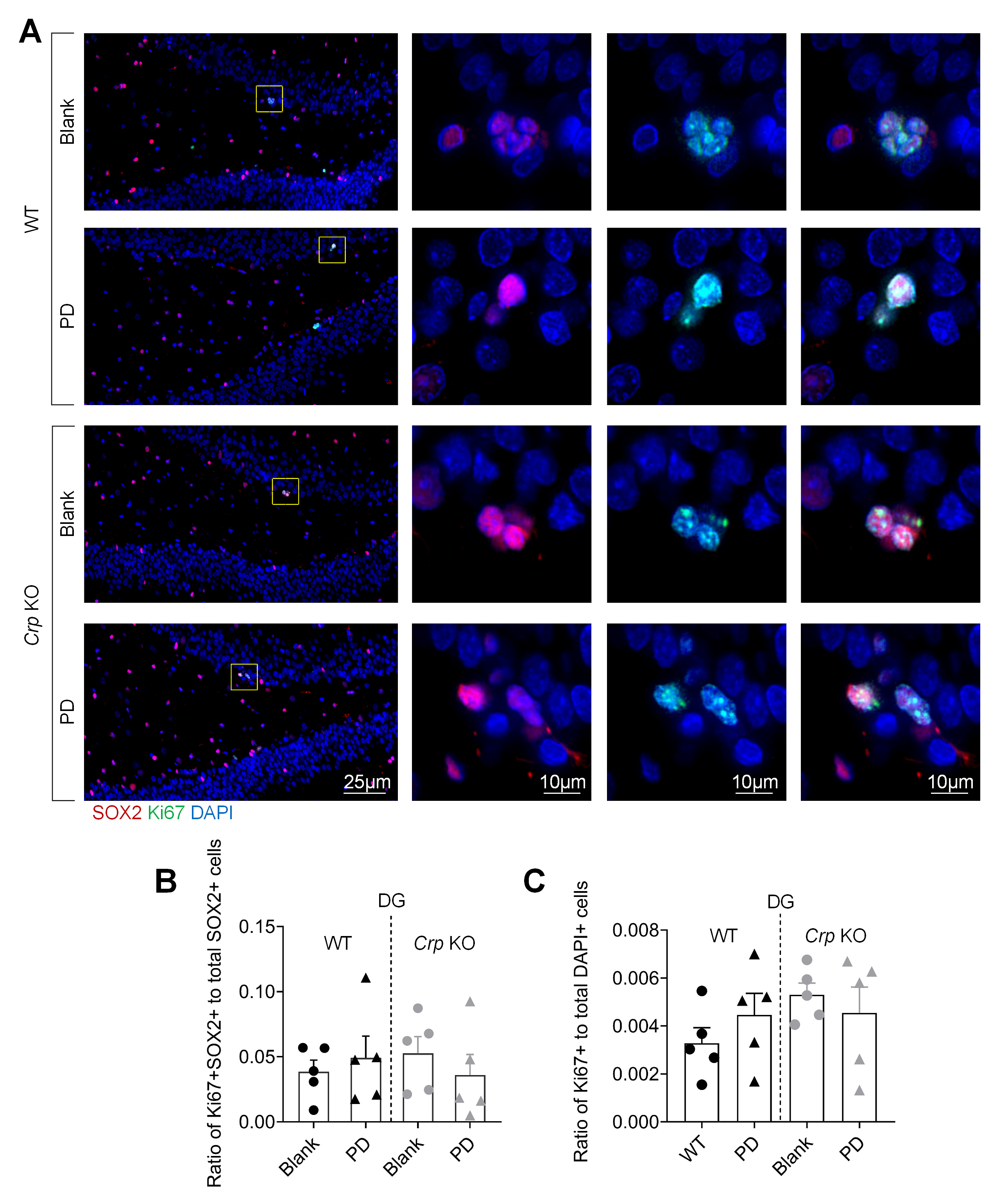


**Figure S8.** NPC proliferation in the DG area of WT and *Crp* KO rats with or without PD. A) Representative IHC images showing the NPC marker SOX2^+^ (red) and the proliferation marker Ki67^+^ (green). DAPI (blue) marks the nuclei. B) Proportion of proliferating NPCs (Ki67⁺SOX2⁺ cells among total SOX2⁺ cells). C) Percentage of Ki67^+^ cells (Ki67⁺ cells among total DAPI⁺ cells). Data are presented as mean ± SEM (n = 5 rats per group). No significant differences were observed.

**Supplementary Table**

**Table S1.** Primer sequences for real-time qPCR

| Gene name |  | Primer sequence (5' to 3') |
| --- | --- | --- |
| *Gapdh (Rat)* | Forward | CATGGCCTTCCGTGTTCCTA |
|  | Reverse | GCCTGCTTCACCACCTTCTT |
| *Il-1 β (Rat)* | Forward | AGCAGCTTTCGACAGTGAGG |
|  | Reverse | CTCCACGGGCAAGACATAGG |
| *Tnf-α (Rat)* | Forward | CTTCTGTCTACTGAACTTCGGG |
|  | Reverse | CTACGGGCTTGTCACTCG |
| *Bmp4 (Rat)* | Forward | CAGAGCCAACACTGTGAGGA |
|  | Reverse | GGGATGCTGCTGAGGTTAAA |
